# Correction of the auditory phenotype in C57BL/6N mice via CRISPR/Cas9-mediated homology directed repair

**DOI:** 10.1186/s13073-016-0273-4

**Published:** 2016-02-15

**Authors:** Joffrey Mianné, Lauren Chessum, Saumya Kumar, Carlos Aguilar, Gemma Codner, Marie Hutchison, Andrew Parker, Ann-Marie Mallon, Sara Wells, Michelle M. Simon, Lydia Teboul, Steve D. M. Brown, Michael R. Bowl

**Affiliations:** Mary Lyon Centre, MRC Harwell, Harwell, Oxford, OX11 0RD UK; Mammalian Genetics Unit, MRC Harwell, Harwell, Oxford, OX11 0RD UK

## Abstract

**Background:**

Nuclease-based technologies have been developed that enable targeting of specific DNA sequences directly in the zygote. These approaches provide an opportunity to modify the genomes of inbred mice, and allow the removal of strain-specific mutations that confound phenotypic assessment. One such mutation is the *Cdh23*^*ahl*^ allele, present in several commonly used inbred mouse strains, which predisposes to age-related progressive hearing loss.

**Results:**

We have used targeted CRISPR/Cas9-mediated homology directed repair (HDR) to correct the *Cdh23*^*ahl*^ allele directly in C57BL/6NTac zygotes. Employing offset-nicking Cas9 (D10A) nickase with paired RNA guides and a single-stranded oligonucleotide donor template we show that allele repair was successfully achieved. To investigate potential Cas9-mediated ‘off-target’ mutations in our corrected mouse, we undertook whole-genome sequencing and assessed the ‘off-target’ sites predicted for the guide RNAs (≤4 nucleotide mis-matches). No induced sequence changes were identified at any of these sites.

Correction of the progressive hearing loss phenotype was demonstrated using auditory-evoked brainstem response testing of mice at 24 and 36 weeks of age, and rescue of the progressive loss of sensory hair cell stereocilia bundles was confirmed using scanning electron microscopy of dissected cochleae from 36-week-old mice.

**Conclusions:**

CRISPR/Cas9-mediated HDR has been successfully utilised to efficiently correct the *Cdh23*^*ahl*^ allele in C57BL/6NTac mice, and rescue the associated auditory phenotype. The corrected mice described in this report will allow age-related auditory phenotyping studies to be undertaken using C57BL/6NTac-derived models, such as those generated by the International Mouse Phenotyping Consortium (IMPC) programme.

**Electronic supplementary material:**

The online version of this article (doi:10.1186/s13073-016-0273-4) contains supplementary material, which is available to authorized users.

## Background

The most common form of sensory disability in the human population is age-related hearing loss (ARHL), which not only causes communication difficulties, but also is associated with social isolation, depression and reduced physical and cognitive function [1]. ARHL is known to be a complex disorder with both genetic and environmental components. Given the high prevalence of the condition (>60 % of people aged ≥70 years), coupled with an ageing population, there is a drive to elucidate the genes and pathology associated with ARHL, thus enabling the development of potential therapeutic strategies. To date, several major genetic studies have investigated adult hearing function in humans, providing candidate gene sets for ARHL susceptibility factors [2–6]. However, the lack of genome-wide significance and absence of replication between studies means validation of these candidate ARHL genes in a model organism is required.

The International Mouse Phenotyping Consortium (IMPC) aims to produce knockout mice for every gene in the mouse genome and test each mutant line through a broad-based phenotyping pipeline, in order to elaborate upon the function of every mouse gene [7, 8]. IMPC uses mutant embryonic stem cells developed by the International Knockout Mouse Consortium (IKMC). The mice generated by the IMPC are preserved in repositories and are available to the scientific community. Utilisation of knockout mice generated by this programme would provide a relatively quick and cost-effective way to obtain models for the validation of genes arising from the human ARHL studies, and to assess the role of genes in ARHL.

A main strength of the IKMC and IMPC programmes is that the respective embryonic stem cell resource and knockout mice produced are generated in a single inbred strain background, namely C57BL/6NTac. However, a major genetic impact of the use of C57BL/6N and the related C57BL/6J strain is that they harbour a fixed hypomorphic allele in the *Cadherin23* gene (*Cdh23*^*ahl*^) that causes these mice to exhibit a high-frequency hearing loss by 3–6 months of age that progresses to a profound impairment by 15 months of age [9, 10]. This renders C57BL/6NTac an unsuitable background strain for investigating potential ARHL-causing genes.

Over recent years several technologies have been developed that allow targeting of specific DNA sequences directly in the zygote, e.g. zinc-finger nuclease (ZFN), transcription activator-like effector nuclease (TALEN), and clustered regularly interspaced short palindromic repeats (CRISPR/Cas9). These are nuclease-based approaches which generate DNA double-stranded breaks (DSBs) at user-defined genomic sequences. The presence of a DSB initiates a repair mechanism that typically leads to non-homologous end joining (NHEJ), resulting in insertion or deletion (indels) events at the targeted locus. However, if the nucleases are used in conjunction with a donor DNA sequence carrying the desired insert with flanking homology to the targeted region, integration by homology directed repair (HDR) can occur. Recently, Low et al. [11] successfully corrected the *Crb1*^*rd8*^ mutation directly in C57BL/6N zygotes using a TALEN-mediated HDR approach, showing recovery of a normal retinal phenotype in heterozygous repaired animals.

Here, we describe the use of targeted CRISPR/Cas9-mediated HDR to correct the *Cdh23*^*ahl*^ allele directly in C57BL/6NTac zygotes. Using two different designs, both employing offset-nicking Cas9 (D10A) nickase with paired RNA guides and a single-stranded oligonucleotide (ssODN) as donor template, we show that allele repair was successfully achieved.

Importantly, we demonstrate that unlike inbred C57BL/6NTac mice (*Cdh23*^*ahl/ahl*^), the heterozygous *Cdh23* repair mice (*Cdh23*^*ahl/753A>G*^) have normal hearing thresholds and a full complement of cochlear sensory hair cell stereocilia bundles at 36 weeks of age. Thus, the repaired C57BL/6NTac mice described here provide an enhanced defined genetic background in which IMPC knockout mouse models can be generated, which are suitable for both assessment of age-related auditory function and age-related behavioural studies that utilize acoustic stimuli as part of the test paradigm.

## Methods

### Mice

All animals were housed and maintained in the Mary Lyon Centre, MRC Harwell under specific opportunistic pathogen-free (SOPF) conditions, in individually ventilated cages adhering to environmental conditions as outlined in the Home Office Code of Practice. All animal studies were licensed by the Home Office under the Animals (Scientific Procedures) Act 1986 Amendment Regulations 2012 (SI 4 2012/3039), UK, and additionally approved by the Institutional Ethical Review Committee. Mice were euthanized by Home Office Schedule 1 methods.

### CRISPR/Cas plasmid generation

#### Oligonucleotide sequences and quality control of constructs

The sequences of all single-stranded oligonucleotides are presented in Additional file 1: Table S1. All constructs were checked using Sanger sequencing (SourceBioscience).

##### Construction of p_1.1 plasmid backbone

A pair of ssODNs containing two unique restriction sites, *Stu*I and *Afl*II (T7GibsonF and T7GibsonR, Integrated DNA Technologies) was annealed and cloned into linearised gRNA_Cloning Vector (Addgene, 41824; George Church, Harvard) digested with *Pvu*I and *Afl*II, using Gibson Assembly Master Mix (New England BioLabs (NEB)). As a result, the new restriction sites have been integrated between the T7 promoter and the single guide RNA (sgRNA) backbone sequence, and can be used to linearise the plasmid for further cloning of chosen protospacer sequences.

##### Construction of p_1.3_D10A plasmid

Cas9 mutant D10A nickase sequence was PCR amplified from pX335 plasmid (Addgene, 42335; Feng Zhang, MIT) using high fidelity Expand Long Range dNTPack (Roche) and oligonucleotides Cas9f4gibson/Cas9r4gibson (Sigma-Aldrich). The PCR product was cloned using Gibson Assembly Master Mix (NEB) into linearised p_1.1 plasmid digested with *Stu*I and *Afl*II in order to express Cas9 mutant D10A under the T7 promoter.

##### Construction of p_1.1_sgRNA plasmids

For each sgRNA (Cdh23_U1, U2 and D1), a pair of ssODNs (Sigma-Aldrich; forward 5′-TAATACGACTCACTATAGG-protospacer-3′; reverse 5′-GACTAGCCTTATTTTAACTTGCTATTTCTAGCTCTAAAAC-protospacer antisense-3′) was hybridised and cloned using Gibson Assembly Master Mix (NEB) into linearised p_1.1 plasmid digested with *Stu*I and *Afl*II in order to express sgRNAs under the T7 promoter.

### In vitro RNA transcription

Cas9 mutant D10A nickase mRNA and sgRNAs were in vitro transcribed from linear forms of p_1.3_D10A and p_1.1_sgRNA plasmids, respectively. Plasmids were linearised with *Xba*I and phenol-chloroform purified. mRNA was synthesised using Message Max T7 Arca Capped Message Transcription Kit (Cellscript) and poly-adenylated using poly(A) polymerase Tailing Kit (Epicentre). Single-stranded guide RNAs were synthesised using MEGAshortscript (Ambion).

RNAs were purified using MEGAclear kit (Ambion). RNA quality was assessed using a NanoDrop (Thermo Scientific) and by electrophoresis on 2 % agarose gel containing Ethidium Bromide (Fisher Scientific).

### In vitro evaluation of sgRNA efficacy

In vitro efficacy of each in vitro transcribed sgRNA (U1, U2 and D1) was assessed using the Guide-it^TM^ sgRNA Screening kit (Clontech) following the manufacturer’s instructions. Analysis of the enzymatic digestions were analysed by electrophoresis on 1.5 % agarose gel containing ethidium bromide (Fisher Scientific).

### Pronuclear microinjections of zygotes

Pronuclear microinjection was performed as per Gardiner and Teboul [12], employing a FemtojJet (Eppendorf) and C57BL/6NTac embryos. Specifically, injection pressure (Pi) was set between 100 and 700 hPa, depending on needle opening; injection time (Ti) was set at 0.5 seconds and the compensation pressure (PC) was set at 10 hPa.

Microinjection buffer (MIB; 10 mM Tris–HCl, 0.1 mM EDTA, 100 mM NaCl, pH7.5) was prepared and filtered through a 2 nm filter and autoclaved. Cas9 mutant D10A nickase mRNA, sgRNAs and ssODNs were diluted and mixed in MIB to the working concentrations of 200 or 100 ng/μl, 100 or 50 ng/μl each and 40 or 20 ng/μl, respectively. Injected embryos were re-implanted in CD1 pseudo-pregnant females. Host females were allowed to litter and rear F_0_ progeny.

### Genotyping

Genomic DNA from F_0_ and F_1_ animals was extracted from ear clip biopsies using a DNA Extract All Reagents Kit (Applied Biosystems). The targeted region was PCR amplified using high fidelity Expand Long Range dNTPack (Roche) and genotyping primers Geno_Cdh23_F1/R1 or F2/R2. PCR products were further purified using a gel extraction kit (Qiagen) and analysed by Sanger sequencing.

PCR products amplified from DNA obtained from F_0_ animals that showed mixed sequencing traces were sub-cloned using a Zero-Blunt PCR cloning Kit (Invitrogen) and 12–24 clones per founder were analysed by Sanger sequencing.

### Detection of ‘off-target’ sequence variations

Genomic DNA was prepared from the spleen of the F_0_ mouse that gave rise to the ‘repaired’ line that underwent auditory phenotyping. Phenol-chloroform extracted DNA was assessed using a NanoDrop (Thermo Scientific), Epoch Microplate Spectrophotometer (Bio-Tek) and by electrophoresis on 1.5 % agarose gel containing ethidium bromide (Fisher Scientific). Whole genome sequencing (WGS) was performed using an Illumina HiSeq 2000 Sequencer. The sequence was mapped using: the BWA-mem aligner with default parameters, v.0.7.10; mouse genome reference mm10 from UCSC (original GRCm38 from NCBI, January 2012); base Phred quality cutoff, NA; keep duplicate reads, no; variable read length support, yes; and, realign gaps, no. Sequence variants were called by The Genome Analysis Toolkit (GATK) [13]. The BAM file was realigned for indel calling by Indel realigner. Indels were called by GATK’s HaplotypeCaller and single nucleotide variants (SNVs) were called by GATK’s UnifiedGenotyper, both using dbSNP version 137 as the background single nucleotide polymorphism (SNP) set. SNV annotations were done using NGS-SNP [14]. Indels were annotated using The Variant Effect Predictor [15].

SNVs and indels were then compared against the precompiled list found in 18 inbred strains from the Mouse Genome Project [16]. Further filtering was done by comparing the novel sequence variations with a wild-type (WT) C57BL/6NTac mouse genome and other in-house mouse sequences. Only high confidence sequence variations were considered in this study. High confidence SNVs and indels refer to those with Phred base quality >150 and read depth >3.

Coding SNVs were investigated by PCR amplification (primers are listed in Additional file 1: Table S1) and Sanger sequencing of DNA from the F_0_ and four C57BL/6NTac WT animals, including that of a stud male employed to produce the microinjected embryos.

### Analysis of the sgRNA predicted off-target sites

Genomic DNA from F_1_ animals was extracted from ear clips using a DNA Extract All Reagents Kit (Applied Biosystems). Potential off-target sites predicted by the WTSI Genome Editing (WGE) webtool for sgRNA_U1 and sgRNA_D1 (design 1), and containing ≤3 mismatches (Additional file 1: Table S2) were PCR amplified using High fidelity Expand Long Range dNTPack (Roche) and the corresponding genotyping primers (Additional file 1: Table S3). PCR amplicons were gel-purified (QIAGEN) and analysed by Sanger sequencing.

### Copy counting for ssODN_U1 in the F_0_ founder and F_1_ progeny

Genomic DNA from F_0_ and F_1_ animals was extracted from ear clips using DNA Extract All Reagents Kit (Applied Biosystems). Reaction mixtures (20 μl) contained 1 μl crude DNA lysate, 1× ddPCR Supermix for probes (Bio-Rad), 225 nM of each primer (two primers per assay used; Additional file 1: Table S1) and 50 nM of each probe (one VIC-labeled probe for the reference gene assay and one FAM-labeled Taqman assay specific to the ssODN_U1 sequence, designed by Biosearch Technologies). These were loaded into the Bio-Rad QX200 AutoDG and droplets generated as per the manufacturer’s instructions. After droplet generation, the oil/reagent emulsion was transferred to a 96-well plate (Eppendorf) and the samples were amplified (95 °C for 10 min, followed by 40 cycles of 94 °C for 30 s and 58 °C for 60 s, with a final elongation step of 98 °C for 10 min). The plate containing the droplet amplicons was analysed as a CNV2 experiment in a QX200 Droplet Reader (Bio-Rad), with channel 1 as unknown and channel 2 as the two-copy reference assay. Standard reagents and consumables supplied by Bio-Rad were used, including cartridges and gaskets, droplet generation oil and droplet reader oil. Analysis was performed using Quantasoft software (Bio-Rad) and copy number determined using a minimum of 10,000 partitions for each sample.

### Auditory-evoked brainstem response

Auditory-evoked brainstem response (ABR) testing was performed as previously described by Hardisty-Hughes et al. [17]. Briefly, mice were anaesthetised using a mixture of ketamine and xylazine and placed on a heated mat in an acoustic chamber (ETS-Lindgren). Broadband click and tone-burst stimuli (8 kHz, 16 kHz, and 32 kHz) were presented free field to the right ear of the mouse. TDT system III hardware and software (Tucker Davis Technology) were used for stimulus presentation and response averaging, starting at the highest level (90 dB sound pressure level (SPL)) and reducing in 5 or 10 dB increments until no response trace could be observed. Mice that displayed no response to a 90 dB SPL stimulus were recorded as 100 dB SPL for subsequent analysis. Recovery of anaesthetized mice was assisted by Atipamezole.

### Cochlear scanning electron microscopy

Animals were euthanized and excised inner ears were fixed overnight in 2.5 % gluteraldehyde in 0.1 M phosphate buffer (Sigma-Aldrich). Fixed ears were decalcified for 48 hours in 4.3 % EDTA in 0.1 M phosphate buffer (Sigma-Aldrich). Fine dissection was performed to reveal the organ of Corti, before osmium tetroxide (Agar Scientific)–thiocarbohydrazide (Fluka) processing (adapted from Hunter-Duvar [18]) was carried out. The inner ears were then dehydrated through increasing strength ethanol solutions (Fisher Scientific) and critical point dried using an Emitech K850 (EM Technologies Ltd). The specimens were then mounted on stubs using silver paint (Agar Scientific) and sputter coated with platinum using a Quorum Q150R S sputter coater (Quorum Technologies). Prepared cochlea were visualised with a JEOL LSM-6010 (Jeol Ltd) scanning electron microscope. Hair cell stereocilia bundle counts were performed by counting the number of adjacent inner hair cells (IHCs) and outer hair cells (OHCs) to ten pillar cells. For the analysis the cochlea was divided into three separate regions (turns), apical (<180° from apex), mid (180–450° from apex), and basal (450–630° from apex). Four ears (one ear per mouse) were analysed for each genotype at each region.

## Results and discussion

### Strategy for *Cdh23*^*ahl*^ allele correction

The *Cdh23*^*ahl*^ allele is a synonymous SNP affecting the last nucleotide of the seventh coding exon of the *Cdh23* gene (c.753). The presence of an adenine (A) rather than a guanine (G) at this position leads to an increased frequency of exon 7 skipping, predisposing inbred mouse strains carrying the A allele to age-related hearing loss [19]. Our strategy for correcting this allele involved directly injecting CRISPR reagents into one-cell stage mouse embryos. This approach has previously been shown to introduce subtle modifications into the genome at high efficiency [20, 21]. Given reported concerns regarding the specificity of the Cas9 nuclease and potential off-target effects, we opted to use paired offset guides along with a nickase version (D10A) of the Cas9 protein to correct the B6N.*Cdh23*^*ahl*^ allele [22, 23].

Employing the Zhang Lab CRISPR design tool (http://crispr.mit.edu/), three protospacers (sgRNA_U1, sgRNA_U2 and sgRNA_D1) were selected allowing for two different designs (design 1, U1/D1, +42 nucleotide offset; and design 2, U2/D1, +13 nucleotide offset) (Fig. 1). The criteria for protospacer sequence selection was based on the following previously suggested recommendations: double nicking resulting in 5′ overhang; low potential off-target effects; as close as possible to the targeted nucleotide; and a short offset [22, 24]. In vitro assessment of these sgRNAs revealed that sgRNA_U1 and sgRNA_D1 both have a high efficacy for directing Cas9 nuclease activity (85 % and 88 % cleavage, respectively), whereas sgRNA_U2 has a lower efficacy (30 %) (Additional file 1: Fig. S1). As such, while design 1 employs two very efficient sgRNAs, the large offset will likely reduce the overall efficiency of this design. In comparison, for design 2 the offset distance is within the optimum range (−4 to +20), but it employs a less efficient sgRNA that will reduce the overall efficiency of this second design [22]. Each design required a specific 121 bp ssODN to be synthesised with the desired correction (A > G) at their centre; these act as DNA donor templates for HDR. In addition to the *Cdh23*^*ahl*^ allele correction, both ssODN templates also contain substitutions in the region complementary to the protospacer sequences (AG > TC in ssODN_U1 and C > T in ssODN_U2). These additional substitutions were added to prevent further modification by the CRISPR/Cas9 post HDR repair. In design 1, the AG > TC substitution does not change the encoded residue (*AG*T > *TC*T, Ser > Ser), and in design 2 the C > T substitution is within the intron (Fig. 1).

**Fig. 1:**
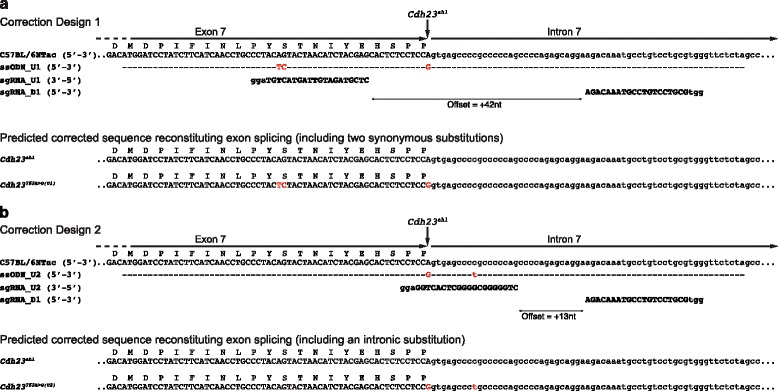
Design of two targeting strategies to recover normal splicing/function of the C57BL/6NTac *Cdh23* gene. **a** Design 1 utilises a 121 bp single-stranded oligonucleotide donor (*ssODN_U1*) in combination with two single guide RNAs (*sgRNA_U1* and *sgRNA_D1*), which flank the *Cdh23* ^*ahl*^ allele. The full ssODN_U1 donor sequence is shown below the C57BL/6NTac *Cdh23* ^*ahl*^ sequence (*uppercase* denotes exonic sequence and *lowercase* denotes intronic sequence), with homology indicated by *dashed lines*, the *Cdh23* ^*ahl*^ allele with an *arrow*, and two base changes shown in *red text*. The two synonymous base substitutions are designed to prevent further modification by the CRISPR/Cas9 following repair. The corrected *Cdh23* ^*753A>G*^ allele is the allele found in inbred mouse strains that do not demonstrate age-related hearing loss (ARHL). The final corrected *Cdh23* ^*753A>G(U1)*^ gene sequence closely matches that found in these non-ARHL inbred strains at the nucleotide level, except for the two synonymous base substitutions (c.724A > T and c.725G > C; *red text*). The *Cdh23* ^*753A>G(U1)*^ product is predicted to be identical to the Cdh23 protein found in non-ARHL mouse strains. **b** Design 2 also utilises a 121 bp ssODN (*ssODN_U2*) in combination with two single guide RNAs, sgRNA_D1 also used in design 1 and sgRNA_U2, which lies across the *Cdh23* ^*ahl*^ locus. The full ssODN_U2 donor sequence is shown below the C57BL/6NTac *Cdh23* ^*ahl*^ sequence, with homology indicated by *dashed lines*, the *Cdh23* ^*ahl*^ allele with an *arrow*, and one intronic base change shown in *red text*. The intronic base substitution is designed to prevent further modification by the CRISPR/Cas9 following repair. The final corrected *Cdh23* ^*753A>G(U2)*^ gene sequence is identical to that found in non-ARHL inbred strains at the nucleotide level, with only an intronic base substitution (c.753 + 9c > t; *red text*). The *Cdh23* ^*753A>G(U2)*^ product is predicted to be identical to the Cdh23 protein found in non-ARHL mouse strains

### Correction of the *Cdh23*^*ahl*^ allele

For each experimental design, in vitro transcribed Cas9 (D10A) nickase mRNA, two sgRNAs and one ssODN were co-injected into one-cell-stage mouse embryos. To optimise the CRISPR/Cas9-mediated HDR, we varied the concentration of these reagents such that we used a ‘low’ and a ‘high’ concentration for each of the two designs (Table 1). For design 1, the percentage of survival (born/injected) was lower when using the higher concentration. However, this likely reflects the smaller number of embryos injected with the lower concentration, as no difference in survival was noted for design 2 when altering concentration (Table 1). Using design 1, a total of 244 embryos were injected over three microinjection sessions giving rise to 72 live founder (F_0_) pups (29.5 %). Whereas, using design 2 a total of 212 embryos were injected over two microinjection sessions, giving rise to 32 live F_0_ pups (15.1 %).

**Table 1:** Number of injected embryos, embryos transferred, pups born, and mutation rate

Design	Cas9 (D10A) mRNA [conc] ng/μl	sgRNAs [conc] ng/μl (each)	ssODN [conc] ng/μl	Number of injected embryos	Lysed after injecting	Lysed (%)	Number of ET	Number of pups born	Born/injected (%)	Number of TG mice	Mutation rate (%)	Number of correct F_0_ mice	Legitimate repair rate (%)
1	100	50	20	24	3	12.5	21	11	45.8	1	9.1	0	0
	200	100	40	220	20	9.1	200	61	27.7	10	16.4	2	3.3
2	100	50	20	101	11	10.9	90	16	15.8	0	0.0	0	0
	200	100	40	111	4	3.6	107	16	14.4	4	25.0	2	12.5
				456	38	8.3	418	104	22.8	15	14.4	4	3.8

*ET* embryos transferred, *TG* transgenic

Generation of mice carrying the *Cdh23* ^*753A>G*^ repair using pronuclear injection of CRISPR/Cas9 reagents. For designs 1 and 2, two sets of injections were performed using either 100, 50, 50 and 20 ng/μl or 200, 100, 100 and 40 ng/μl of Cas9 (D10A) nickase mRNA, sgRNAs_U, sgRNA_D and ssODN, respectively. Higher efficiency was obtained when using the higher concentrations. The percentages of mutation rate and legitimate repair rate have both been calculated using the number of pups born as the denominator

To identify CRISPR-mediated events within the 104 F_0_ pups, DNA extracted from ear biopsies was utilised for PCR amplification and Sanger sequencing of the targeted locus. Animals with a complex genotype (i.e. mosaic with two or more mutated alleles) or potentially harbouring the correctly repaired allele were further characterised by sub-cloning and Sanger sequencing of the targeted region. Of the 72 F_0_ pups generated using design 1, 11 were found mutated on-target, including two containing the repaired *Cdh23*^*753A>G*^ allele. For design 2, 4 of the 32 F_0_ pups were found mutated on-target, including two containing the repaired *Cdh23*^*753A>G*^ allele. Interestingly, in addition to the repaired *Cdh23*^*753A>G*^ allele, these animals were also carrying alleles incorrectly repaired through HDR (which we called “illegitimate repair”) (Fig. 2). Thus, from a total of 456 injected embryos we recovered 104 pups (22.8 %), 15 of which are transgenic (14.4 %), including four carrying the correct *Cdh23*^*753A>G*^repair (3.8 %) (Table 1). Interestingly, 14 of the 15 transgenic mice (including the four with the repaired allele) were obtained from microinjections using the higher concentration of CRISPR/Cas9 reagents, while only one transgenic mouse was produced using the lower concentration.

**Fig. 2:**
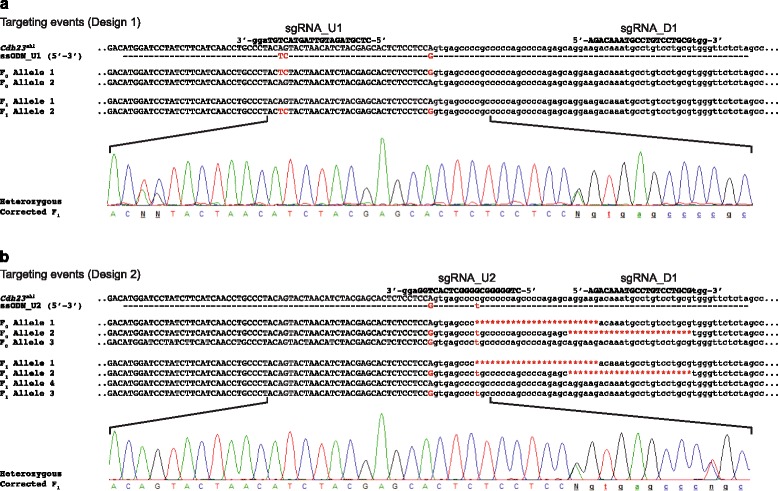
Targeting events in F_0_ mice and transmission to F_1_ offspring. **a** Targeting events in an F_0_ using design 1. The genotype of the F_0_ was directly assessed by ear clip DNA analysis. This revealed the presence of two alleles: allele 1 corresponding to the legitimate HDR event and allele 2 corresponding to the WT allele. The genotype of the F_0_ animal was confirmed through the transmission of both alleles to the F_1_ population, several of which were identified as heterozygous for the legitimate repair (see trace). **b** Targeting events in an F_0_ using design 2. The genotype of the F_0_ was directly assessed by ear clip DNA analysis. This revealed the presence of three alleles: allele 1 corresponding to an NHEJ event consisting of a 24 nucleotide deletion; allele 2 corresponding to a combination of the intended repair and additional sequence changes (illegitimate repair) comprising modification of the two targeted nucleotides and a 3′ deletion of 24 nucleotides; and allele 3 corresponding to a legitimate repair. The genotype of the F_0_ animal was confirmed through transmission of these three alleles to the F_1_ population (F_1_ allele 1 and F_1_ allele 2). However, one additional allele was also identified in the F_1_ population: ‘F_1_ allele 4’ comprising WT sequence

The four F_0_ mice identified as having the correctly repaired *Cdh23*^*753A>G*^ allele were shown to be highly mosaic at the target region. To further characterise the alleles present in these mice, PCR amplification of the targeted locus was undertaken, and the resulting amplicons were sub-cloned and Sanger sequenced. This confirmed that the four F_0_ mice had undergone CRISPR/Cas9-mediated repair of the *Cdh23*^*ahl*^ allele, all showing legitimate repair sequences (Fig. 2). Going forward, this experimental design can be employed to repair the *Cdh23*^*ahl*^ allele in other mouse strains, e.g. C57BL/6J.

### Germline transmission

To establish heritability of the repaired *Cdh23*^*753A>G*^ allele and to segregate the alleles detected in mosaic founders, three of the *Cdh23*^*753A>G*^ F_0_ mice were bred to stock C57BL/6NTac mice.

Analysis of the F_1_ offspring obtained from each of these three founders revealed transmission of the repaired *Cdh23*^*753A>G*^ allele (Fig. 2a).

However, analysis of the F_1_ generation also demonstrated that alleles represented in F_0_ somatic cells (e.g. an ear biopsy) can be absent, or under-represented, in F_0_ germ cells, as determined by their non-transmission to the F_1_ progeny. In addition, the reciprocal can occur where alleles not detected within the somatic cells of a F_0_ mouse are identified in their F_1_ progeny. This latter case was observed for both of our design 2 founder mice, which transmitted an additional WT allele not previously detected at the F_0_ stage (Fig. 2b). These data highlight three important issues associated with CRISPR-aided mutagenesis: firstly, while F_0_ mice may give initial insight into phenotypic outcomes of CRISPR/Cas9 targeting, they are genotypically unpredictable due to potential mosaicism — therefore, more detailed studies should be undertaken using ≥ F_1_ animals; secondly, analysis of F_1_ mice may identify ‘hidden’ alleles not seen in the somatic cells of F_0_ mice; and thirdly, it is important to sequence the flanking regions when producing point mutations by CRISPR-aided mutagenesis to confirm that targeted mutations are not associated with unwanted indels.

### Analysis of predicted off-target sites

A major concern within the research community regarding CRISPR/Cas9 technology is the potential for ‘off-target’ events to occur, as these could cause deleterious/confounding phenotypic traits [25, 26]. In order to increase the specificity of our system we decided to use the Cas9 mutant (D10A) double nicking system, which has been proposed to reduce the likelihood of ‘off-target’ mutations [24]. Moreover, the sgRNAs used in this study were specifically selected as they show very few potential off-target sites, particularly on chromosome 10, which could not be easily segregated out through breeding, unlike off-target mutations on other chromosomes. Potential off-target binding sites for the design 1 guides were determined using the WTSI Genome Editing tool [27]. When allowing up to four nucleotide mismatches, 55 and 173 off-target sites were predicted for sgRNA_U1 and sgRNA_D1, respectively (Additional file 1: Table S2). To assess the possibility of off-target Cas9-mediated damage, WGS was performed for the F_0_ used to establish the line that underwent phenotyping as part of this study. From approximately 152 million paired-end reads (150 bp), the alignment had a 9× average read depth with 1.5 % assembly gaps. To enable the identification of CRISPR/Cas9-induced variants we also sequenced a WT C57BL/6NTac from our breeding colony. Sequence variants found to be common between the CRISPR/Cas9 F_0_, WT C57BL/6NTac, and those variants present in public repositories (including the Mouse Genomes Project and dbSNP [16, 28]) were eliminated from further analysis. Using a standard mutation detection tool we searched for potential sequence variations (SNVs and small indels) in the predicted off-target sites and surrounding the on-target site. No putative SNVs or indels were detected at any of the 228 predicted off-target sites examined (Table 2). Due to the potential of mosaicism within the F_0_ mouse to confound the WGS data, we also assessed the F_1_ progeny for the presence of ‘hidden’ off-target damage transmitted through the germline. We amplified and Sanger sequenced the 14 most closely related off-target sites (three or fewer mismatches) predicted for sgRNA_U1 and sgRNA_D1 (Additional file 1: Table S3). No sequence variants were identified, confirming the high specificity of the double nicking system. In addition to the *Cdh23*^*753A>G*^ repair edit, two additional genome edits were also added as part of design 1 (Fig. 1 and Table 3). All three edits were detected by WGS. To investigate the coding (missense, stop gain/loss and splice) mutation frequency in the F_0_ repaired *Cdh23*^*ahl/753A>G*^ genome, we first used an automated SNV detection pipeline. This identified 42 potential coding SNVs. Subsequently we manually compared these with the WT C57BL/6NTac sequence and other mouse strains from our in-house sequence library. We found the majority of these coding SNVs (41) were present at low allele frequencies in more than two sequences or in regions with misaligned reads, and so were eliminated from further analysis. The one remaining coding SNV and six predicted false positives (randomly selected) were assessed using Sanger sequencing (Table 3). Of these: four SNVs were found not to be present in the repaired *Cdh23*^*ahl/753A>G*^ sequence and therefore confirmed as false positives; one SNV (Ubox5) was found to be present in both the WT C57BL/6NTac sequence and the repaired *Cdh23*^*ahl/753A>G*^ sequence; one SNV remained undetermined due to the repetitive nature of the genomic locus (*Vmn2r114*); and one SNV (*Fam184b*) was found only in the repaired *Cdh23*^*ahl/753A>G*^ sequence. Sanger sequencing of additional C57BL/6NTac mice from our WT stock identified that the *Fam184b* SNV is heterogeneously present, indicating that this mutation has recently arisen spontaneously within the colony (less than ten generations, as the WT C57BL/6NTac colony is restocked every ten generations from the supplier, Taconic Biosciences). This allele on chromosome 5 will be easily segregated from the repaired *Cdh23*^*ahl/753A>G*^ allele located on chromosome 10. Importantly, no novel coding small indels were predicted in the repaired *Cdh23*^*ahl/753A>G*^ genome.

**Table 2:** Summary of predicted off-target sites for sgRNA_U1 and sgRNA_D1, and off-target variations identified

Predicted off-target sites	With two mismatches	With three mismatches	With four mismatches	Number of off-target SNVs and indels
Exonic	0	0	8	0
Intergenic	0	6	122	0
Intronic	2	6	82	0

Summary of the predicted off-target sites with four or fewer mismatches for both sgRNA_U1 (total of 55 sites) and sgRNA_D1 (total of 173 sites) separated into genic type. Whole genome sequence analysis of the founder F_0_ mouse, used to establish the line that was phenotyped in this study, demonstrated no modifications to be present at these 228 predicted off-target sites

**Table 3:** Novel high-confidence coding SNVs identified in the F_0_ repaired *Cdh23* ^*ahl/753A>G*^ genome

Chr	Position	B6J Ref	F_0_ SNV	Functional annotation	Gene	AA position	Reference AA	Alternative AA	Validated
2	130591861	C	T	missense_variant	Ubox5	522	C	Y	Present^a^
5	45582909	G	A	missense_variant	*Fam184b*	312	R	W	Present
8	35482596	C	T	missense_variant	*Eri1*	136	E	K	Not present
8	35482601	G	A	missense_variant	*Eri1*	134	T	I	Not present
8	35482602	T	G	missense_variant	*Eri1*	134	T	P	Not present
10	60530975	C	G	missense_variant	*Cdh23*	242	S	T	Present^b^
10	60530976	T	A	missense_variant	*Cdh23*	242	S	C	Present^b^
11	69826746	A	C	splice_region_variant	*Nlgn2*	544	T	T	Not present
17	23310616	G	T	missense_variant	*Vmn2r114*	171	P	T	UD

*Chr* chromosome, *B6J Ref* C57BL/6J reference genome sequence, *F*_*0*_ *SNV* founder-identified single nucleotide variant, *AA* amino acid, *UD* undetermined due to the repetitive nature of the sequence encompassing the SNV

^a^Present in the WT C57BL/6NTac strain

^b^These two nucleotide changes were specifically introduced as part of correction design 1, and when both are present led to a synonymous change (p.S242S)

Another reported concern regarding genome editing is the potential for the donor ssODN to randomly incorporate at strand breaks in the genome. To address this possibility we interrogated the F_0_ WGS data, which only identified the ssODN_U1 sequence at the on-target site. In addition, copy counting of the ssODN_U1 using Droplet Digital™ PCR was undertaken for this F_0_ animal and its F_1_ progeny. This confirmed that the ssODN has only incorporated once within the genome of our repaired mice.

### Correction of C57BL/6N auditory phenotype

The age-related hearing loss observed in C57BL/6 mice (B6J and B6N) has been extensively characterized [10]. The *Cdh23*^*ahl*^ hearing loss susceptibility allele (c.753A) carried by these inbred strains cause the mice to develop a high-frequency hearing loss by 3 to 6 months of age that progresses to a profound impairment by 15 months of age. Consistent with the exhibited auditory decline, age-related histopathological changes occur within the ageing cochleae of C57BL/6 mice. It has been shown that loss of sensory hair cell stereocilia bundles begins in the base of the cochlea (the region of the cochlea that detects high-frequency sound) and gradually spreads apically (the region that detects low-frequency sound) with advancing age. While the loss includes both inner and outer hair cell bundles, the loss of outer hair cell bundles precedes and is more extensive than inner hair cell bundle loss [9].

Hearing assessment by ABR threshold analysis was undertaken for wild-type C57BL/6NTac (*Cdh23*^*ahl/ahl*^) mice and heterozygous repaired C57BL/6NTac (*Cdh23*^*ahl/753A>G*^) littermates (*Cdh23*^*ahl/753A>G*^) at 24 and 36 weeks of age. Figure 3 shows the ABR threshold means for each auditory stimulus. By 24 weeks of age the *Cdh23*^*ahl/ahl*^ mice (*n* = 17) already have significantly elevated hearing thresholds (>60 dB SPL) at the highest frequency tested (32 kHz), whereas their lower frequency (8 and 16 kHz) hearing thresholds are within the normal range (15–35 dB SPL). However, at 24 weeks of age the *Cdh23*^*ahl/753A>G*^ mice (*n* = 13) have hearing thresholds within the normal range (15–35 dB SPL) at all frequencies tested (8, 16 and 32 kHz) (Fig. 3a).

**Fig. 3:**
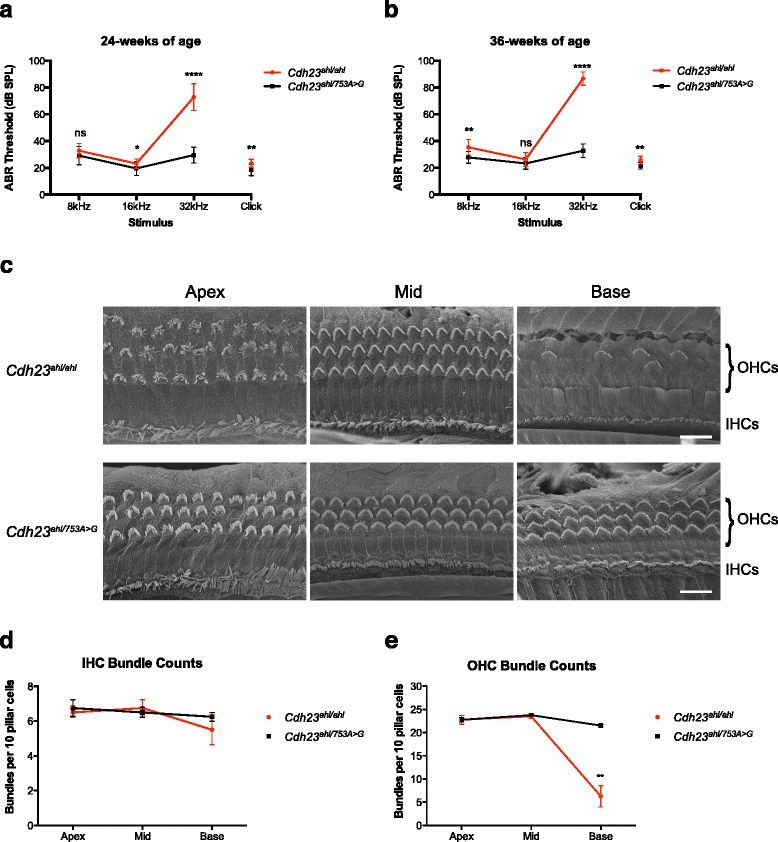
CRISPR-Cas9 mediated repair of the *Cdh23* ^*ahl*^ allele in C57BL/6NTac mice preserves age-related high-frequency hearing and sensory hair cell stereocilia bundles. **a** ABR measurements from 24-week-old C57BL/6NTac mice (*Cdh23* ^*ahl/ahl*^) and their heterozygous ‘repaired’ C57BL/6NTac littermates (*Cdh23* ^*ahl/753A>G*^). As previously reported, by 24 weeks of age the WT *Cdh23* ^*ahl/ahl*^ (*n* = 17) mice show elevated hearing thresholds (>60 dB SPL) for the 32 kHz stimulus, the highest frequency tested. However, at 24 weeks of age the *Cdh23* ^*ahl/753A>G*^ (*n* = 13) littermates do not have elevated thresholds at 32 kHz, but instead display thresholds similar to those measured for the 8 and 16 kHz stimuli (~20–35 dB SPL). **b** By 36 weeks of age the *Cdh23* ^*ahl/ahl*^mice (*n* = 12) have very elevated hearing thresholds (≥80 dB SPL) for the 32 kHz stimulus, showing progression of the high-frequency hearing impairment. However, at 36 weeks of age the *Cdh23* ^*ahl/753A>G*^ littermate mice (*n* = 9) still exhibit hearing thresholds within the normal range (~20–35 dB SPL) for all frequencies tested (8, 16 and 32 kHz). These results indicate that the CRISPR/Cas9 repaired *Cdh23* allele is sufficient to preserve high-frequency hearing in C57BL/6NTac mice. ABR data analysed using an unpaired *t* test with Welch’s correction, and shown as mean ± standard deviation. **c** Scanning electron micrographs of the sensory epithelia in the apex, mid and base regions of the cochlea in WT C57BL/6NTac (*Cdh23* ^*ahl/ahl*^) and heterozygous repaired C57BL/6NTac (*Cdh23* ^*ahl/753A>G*^) mice at 36 weeks of age. Loss of outer hair cell (*OHC*) bundles is evident at the cochlear base of *Cdh23* ^*ahl/ahl*^ mice. No loss of OHC bundles is evident in the age-matched *Cdh23* ^*ahl/753A>G*^ littermate mice. **d**, **e** Cochleograms showing the number of inner hair cell (*IHC*) and OHC bundles present in the apex, mid and base regions of the cochlea in WT C57BL/6NTac (*Cdh23* ^*ahl/ahl*^) (*n* = 4) and heterozygous repaired C57BL/6NTac (*Cdh23* ^*ahl/753A>G*^) (*n* = 4) mice at 36 weeks of age. By 36 weeks of age, no significant loss of IHC bundles in any cochlear region of *Cdh23* ^*ahl/ahl*^ or *Cdh23* ^*ahl/753A>G*^ mice is observed. Significant loss of OHC bundles is found at the cochlear base of *Cdh23* ^*ahl/ahl*^ mice, whereas no loss is found in the cochleae of *Cdh23* ^*ahl/753A>G*^ mice. Hair cell count data analysed using an unpaired *t* test with Welch’s correction, and shown as mean ± standard error of the mean. *p < 0.05, **p < 0.01, ****p < 0.0001, *ns* not significant

By 36 weeks of age the *Cdh23*^*ahl/ahl*^ mice (*n* = 12) show an expected increase in hearing threshold at 32 kHz (>80 dB SPL) compared with their 24-week threshold, while their lower frequency hearing thresholds are not greatly increased. At 36 weeks of age the *Cdh23*^*ahl/753A>G*^ mice (*n* = 9) still have hearing thresholds within the normal range (15–35 dB SPL) at all frequencies tested (8, 16 and 32 kHz) (Fig. 3b).

Sensory hair cells were assessed using scanning electron microscopy for *Cdh23*^*ahl/ahl*^ and *Cdh23*^*ahl/753A>G*^ mice at 36 weeks of age. Figure 3c shows micrographs taken of the sensory epithelia from three regions of the cochlea (apex, mid and base). This shows that by 36 weeks of age the *Cdh23*^*ahl/ahl*^ mice have a normal complement of hair cells (one row of IHCs and three rows of OHCs) in the apex and mid regions of the cochlea. However, as expected, they show loss of OHC stereocilia bundles in the base. In contrast, at 36 weeks of age the *Cdh23*^*ahl/753A>G*^ mice do not show loss of IHC or OHC bundles, in any region of the cochlea.

Hair cell bundle counts were undertaken to quantify the number of IHC and OHC stereocilia bundles present in the different cochlear regions of these mice. Figure 3d shows there is no difference in the number of IHC bundles found in *Cdh23*^*ahl/ahl*^ and *Cdh23*^*ahl/753A>G*^ mice at 36 weeks of age. Figure 3e shows that while there is no difference in the number of OHC bundles found in the apex and mid regions of *Cdh23*^*ahl/ahl*^ and *Cdh23*^*ahl/753A>G*^ mice at 36 weeks of age, there is a significant difference in the number of OHC bundles present in the cochlear base. The *Cdh23*^*ahl/ahl*^ mice show a loss of >50 % of their OHC stereocilia bundles, whereas the *Cdh23*^*ahl/753A>G*^ mice have a full complement of OHC bundles at the base of the cochlea.

Together, these data show that CRISPR/Cas9-mediated HDR using ssODN genotypically corrected the *Cdh23*^*ahl*^ allele in C57BL/6NTac mice and phenotypically rescued their age-related auditory function. The repaired allele is being maintained on a C57BL/6NTac background and available from the Frozen Embryo and Sperm Archive (FESA) at MRC Harwell via MouseBook, an integrated portal of mouse resources [29].

## Conclusions

We report the use of offset-nicking CRISPR/Cas9-mediated HDR to efficiently and precisely correct the *Cdh23*^*ahl*^ allele directly in C57BL/6NTac zygotes. Our sequencing data suggest the approach is highly specific, with no lesions identified at any of the predicted off-target sites. Critically, mice heterozygous for the repaired allele maintain normal hearing function, with complete abrogation of both the progressive hearing loss and sensory cell degeneration phenotypes common to the WT C57BL/6NTac strain.

It has previously been reported that nuclease-mediated HDR can cause mosaicism in founder animals, and this was found in our F_0_ mice [30]. The genotypic complexity present in the founder animals precludes definitive phenotypic assessment of these mice and confirms that detailed phenotype data should be acquired from subsequent generations (F_1_ onwards), once the alleles have been segregated and a precise genotype for each animal is known.

The study of mouse mutants, whether induced, engineered, or spontaneous, has been invaluable for the elucidation of genes required for mammalian audition, and remain the model organism of choice. Importantly, characterisation of these models has provided mechanistic insight into the biology of congenital and early-onset hearing loss by elucidating gene function [17, 31]. However, less progress has been made regarding understanding the genetics and pathological processes associated with age-related hearing loss [32]. By crossing to the C57BL/6NTac.*Cdh23*^*753A>G*^ mice generated in this study, mouse models derived from IKMC embryonic stem cells and IMPC-produced knockout mutants can be maintained on an enhanced C57BL/6N background that will permit investigation of auditory function in aged animals, thus providing insight into genes required for age-related hearing. In addition, models maintained on the repaired background can be employed for age-related behavioural studies that utilize acoustic stimuli as part of the test paradigm, such as acoustic startle and pre-pulse inhibition.

## Additional file

Additional file 1:
**The following additional data are available with the online version of this paper.** **Figure S1.** In vitro assessment of sgRNA efficacy. **Table S1.** The sequences of the oligonucleotides used in this study. **Table S2.** The sequences and locations of the predicted off-target sites for the two sgRNAs used in design 1. **Table S3.** Oligonucleotide sequences for Sanger sequencing of sgRNA_U1 and sgRNA_D1 predicted off-target sites (three or fewer mismatches). (DOCX 922 kb)

## Abbreviations

ABR: Auditory-evoked brainstem response
ARHL: Age-related hearing loss
bp: Base pair
CRISPR: Clustered regularly interspaced short palindromic repeats
DSB: DNA double-stranded break
GATK: Genome Analysis Toolkit
HDR: Homology directed repair
IHC: Inner hair cell
IKMC: International Knockout Mouse Consortium
IMPC: International Mouse Phenotyping Consortium
indel: Insertion or deletion
MIB: Microinjection buffer
NEB: New England BioLabs
NHEJ: Non-homologous end joining
OHC: Outer hair cell
PCR: Polymerase chain reaction
sgRNA: Single guide RNA
SNP: Single nucleotide polymorphism
SNV: Single nucleotide variant
SPL: Sound pressure level
ssODN: Single-stranded oligonucleotide
TALEN: Transcription activator-like effector nuclease
WGS: Whole genome sequencing
WT: wild type
ZFN: Zinc-finger nuclease
