# Supplementary material for: Correction of the auditory phenotype in C57BL/6N mice via CRISPR/Cas9-mediated homology directed repair
Source: Genome Med. 2016 Feb 15;8:16. doi: 10.1186/s13073-016-0273-4 (PMC4753642; doi:10.1186/s13073-016-0273-4)
Supplement: Additional file 1: — The following additional data are available with the online version of this paper. Figure S1. In vitro assessment of sgRNA efficacy. Table S1. The sequences of the oligonucleotides used in this study. Table S2. The sequences and locations of the predicted off-target sites for the two sgRNAs used in design 1. Table S3. Oligonucleotide sequences for Sanger sequencing of sgRNA_U1 and sgRNA_D1 predicted off-target sites (three or fewer mismatches). (DOCX 922 kb) [file 13073_2016_273_MOESM1_ESM.docx]

**Additional file 1**

**Correction of the auditory phenotype in C57BL/6N mice via CRISPR/Cas9-mediated homology directed repair**

Joffrey Mianné, Lauren Chessum, Saumya Kumar, Carlos Aguilar, Gemma Codner, Marie Hutchison, Andrew Parker, Ann-Marie Mallon, Sara Wells, Michelle M. Simon, Lydia Teboul, Steve D.M. Brown, Michael R. Bowl

Figure S1.

***In vitro* assessment of sgRNA efficacy.** PCR template, sgRNA and Cas9 nuclease were combined in an *in vitro* cleavage assay. In order to calculate the *in vitro* efficacy of each sgRNA, reactions were electrophoresed on an agarose gel, and the intensity of un-cleaved template compared to that of the un-cut control DNA (U1 and D1 *vs* un-cut control 1, U2 *vs* un-cut control 2). The *in vitro* efficacy is given as the percentage of the product cleaved.

Table S1. Sequence of oligonucleotides

| Primer name | 5'-3' sequence |
| --- | --- |
| T7GibsonF | TGCCGTACGTATAGGCTGCGCAACTGTTGGGAAGGGCGATCGGTGCGGGCCTCTTCGCTATTACGCCAGCTGGCGAAAGGGGGATGTGCTGCAAGGCGATTAAGTTGGGTAACGCCAGGGTTTTCCCAGTCACGACGTTGTAAAACGACG |
| T7GibsonR | TTTTCAAGTTGATAACGGACTAGCCTTATTTTAACTTAAGtatatatataGAAGACatcaggCCTATAGTGAGTCGTATTACAATTCACTGGCCGTCGTTTTACAACGTCGTGACTGGGAAAACCCTGGCGTTACCCAACTTAATCGCCTTGCAG |
| Cas9f4gibson | CCAGTGAATTGTAATACGACTCACTATAGG |
| Cas9r4gibson | TTTTCAAGTTGATAACGGACTAGCCTTATTTTAACTTAAG |
| Cdh23_U1_fw | TAATACGACTCACTATAGGctcgtagatgttagtactgt |
| Cdh23_U1_rev | GACTAGCCTTATTTTAACTTGCTATTTCTAGCTCTAAAACacagtactaacatctacgag |
| Cdh23_D1_fw | TAATACGACTCACTATAGGagacaaatgcctgtcctgcg |
| Cdh23_D1_rev | GACTAGCCTTATTTTAACTTGCTATTTCTAGCTCTAAAACcgcaggacaggcatttgtct |
| Cdh23_U2_fw | TAATACGACTCACTATAGGctgggggcggggctcactgg |
| Cdh23_U2_rev | GACTAGCCTTATTTTAACTTGCTATTTCTAGCTCTAAAACccagtgagccccgcccccag |
| Geno_Cdh23_F1 | TCAGGCTCCCCTGCTTCTAT |
| Geno_Cdh23_R1 | AGCTACCAGGAACAGCTTGG |
| Geno_Cdh23_F2 | CAGGAGAGCTAGTCCTTGGC |
| Geno_Cdh23_R2 | TAGCCCATTTGACCAGGTGC |
| Ngln2_NGS_F | GCAGACCAAGTTGCACCAAA |
| Ngln2_NGS_R | CAACCGGATGGTGAGACCTT |
| Vmn2r114_NGS_F | TCATGTTGACAAAGGCAAAGCA |
| Vmn2r114_NGS_R | ATGGGGTGGAGACAATGCAA |
| Fam184b_NGS_F | GGAACCCCTTTGCACCAAAA |
| Fam184b_NGS_R | TAACTTTCCCAGGGCCAGACT |
| Eri1_NGS_F | GGGGTACTTACGTGCAGCTT |
| Eri1_NGS_R | AGCATTTGTTAGTTTTGCTGTGG |
| Ubox5_NGS_F | TGCAGATAACATGGGGCACA |
| Ubox5_NGS_R | TGCACAGCACCAAGATAGAGA |
| ssODN_U1_F | CTTCATCAACCTGCCCTACTC |
| ssODN_U1_R | ACGCAGGACAGGCATTTGTC |
| ssODN_U1_probe | 5'FAM/CTCTCCTCCGGTGAGC |
| ref_F | GCCCCAGCACGACCATT |
| ref_R | TAGTTGGCATCCTTATGCTTCATC |
| ref_Probe | 5’VIC/CCAGCTCTCAAGTCG/MGB |

Table S2. Predicted off-target sites

| **sgRNA U1** |  |  |  |  |  |
| --- | --- | --- | --- | --- | --- |
| **Location** | **Sequence** | **PAM** | **Mismatches** | **Strand** | **Type** |
| Original CRISPR | CTCGTAGATGTTAGTACTGT | AGG |  |  | Exonic |
| 10:60530959-60530981 | CTCGTAGATGTTAGTACTGT | AGG | 0 | + | Exonic |
| [12:30168821-30168843](http://www.ensembl.org/Mus_Musculus/psychic?q=12:30168821-30168843) | CT**GT**TAGATGTT**T**GTACTGT | TGG | 3 | + | Intergenic |
| [13:37743124-37743146](http://www.ensembl.org/Mus_Musculus/psychic?q=13:37743124-37743146) | **T**TC**A**TAGAT**T**TTAGTACTGT | GGG | 3 | + | Intergenic |
| [16:55439600-55439622](http://www.ensembl.org/Mus_Musculus/psychic?q=16:55439600-55439622) | **T**TC**C**TAGATGTTAG**G**ACTGT | GGG | 3 | - | Intergenic |
| [16:87306283-87306305](http://www.ensembl.org/Mus_Musculus/psychic?q=16:87306283-87306305) | CT**A**GT**T**GA**A**GTTAGTACTGT | TGG | 3 | - | Intergenic |
| [19:15387252-15387274](http://www.ensembl.org/Mus_Musculus/psychic?q=19:15387252-15387274) | CTC**A**TAGATG**A**TAGTA**G**TGT | GGG | 3 | - | Intergenic |
| [1:136148120-136148142](http://www.ensembl.org/Mus_Musculus/psychic?q=1:136148120-136148142) | CTC**A**T**G**GATG**G**T**G**GTACTGT | GGG | 4 | + | Intronic |
| [1:180187862-180187884](http://www.ensembl.org/Mus_Musculus/psychic?q=1:180187862-180187884) | **T**T**G**G**A**AGATGTTAG**C**ACTGT | AGG | 4 | - | Intronic |
| [10:4510066-4510088](http://www.ensembl.org/Mus_Musculus/psychic?q=10:4510066-4510088) | CT**T**G**A**AGATGTTA**A**TACT**T**T | TGG | 4 | + | Intronic |
| [10:91391462-91391484](http://www.ensembl.org/Mus_Musculus/psychic?q=10:91391462-91391484) | CT**GT**TAGATGT**G**AGTACTG**G** | GGG | 4 | + | Intergenic |
| [11:20904545-20904567](http://www.ensembl.org/Mus_Musculus/psychic?q=11:20904545-20904567) | **A**TCGTAGATGT**C**AGTAC**CA**T | GGG | 4 | - | Intergenic |
| [11:36690892-36690914](http://www.ensembl.org/Mus_Musculus/psychic?q=11:36690892-36690914) | C**A**CGTAG**C**T**T**TTAGT**G**CTGT | GGG | 4 | + | Intronic |
| [11:41403458-41403480](http://www.ensembl.org/Mus_Musculus/psychic?q=11:41403458-41403480) | CTC**T**T**TT**ATGTTAGTA**T**TGT | GGG | 4 | - | Intergenic |
| [11:120180051-120180073](http://www.ensembl.org/Mus_Musculus/psychic?q=11:120180051-120180073) | CT**G**G**A**AGAT**C**TTAG**C**ACTGT | GGG | 4 | - | Intergenic |
| [12:11010993-11011015](http://www.ensembl.org/Mus_Musculus/psychic?q=12:11010993-11011015) | C**C**CGTAGA**G**GT**C**AGTACTG**A** | TGG | 4 | - | Intergenic |
| [12:71937201-71937223](http://www.ensembl.org/Mus_Musculus/psychic?q=12:71937201-71937223) | CTC**TG**AGATGTTAG**C**ACT**C**T | AGG | 4 | + | Intronic |
| [13:10017003-10017025](http://www.ensembl.org/Mus_Musculus/psychic?q=13:10017003-10017025) | CT**GT**TAG**C**TGTTAGTAC**A**GT | AGG | 4 | + | Intergenic |
| [13:34166510-34166532](http://www.ensembl.org/Mus_Musculus/psychic?q=13:34166510-34166532) | CT**G**GTAGATGT**C**A**C**TACTG**C** | AGG | 4 | + | Intronic |
| [13:79273433-79273455](http://www.ensembl.org/Mus_Musculus/psychic?q=13:79273433-79273455) | CT**G**GT**T**GATGTTAGT**C**CTG**C** | TGG | 4 | + | Intergenic |
| [14:78199562-78199584](http://www.ensembl.org/Mus_Musculus/psychic?q=14:78199562-78199584) | CTCGT**G**GATGTTAG**C**AC**CT**T | TGG | 4 | - | Intergenic |
| [14:79977075-79977097](http://www.ensembl.org/Mus_Musculus/psychic?q=14:79977075-79977097) | CT**A**GTAGATGTT**G**GT**G**CT**C**T | TGG | 4 | + | Intergenic |
| [14:83136553-83136575](http://www.ensembl.org/Mus_Musculus/psychic?q=14:83136553-83136575) | C**C**C**A**TA**C**ATGTTA**C**TACTGT | TGG | 4 | + | Intergenic |
| [15:40554686-40554708](http://www.ensembl.org/Mus_Musculus/psychic?q=15:40554686-40554708) | **A**T**T**GTAGATGT**C**AGTACT**A**T | GGG | 4 | - | Intergenic |
| [15:95682093-95682115](http://www.ensembl.org/Mus_Musculus/psychic?q=15:95682093-95682115) | CTC**C**T**T**GATG**G**TAGTACTG**A** | TGG | 4 | + | Intergenic |
| [16:12821625-12821647](http://www.ensembl.org/Mus_Musculus/psychic?q=16:12821625-12821647) | **T**T**G**GTAG**T**TG**C**TAGTACTGT | AGG | 4 | - | Intergenic |
| [16:33338370-33338392](http://www.ensembl.org/Mus_Musculus/psychic?q=16:33338370-33338392) | CT**G**GTA**T**A**G**GTTAG**G**ACTGT | AGG | 4 | - | Intergenic |
| [16:97207391-97207413](http://www.ensembl.org/Mus_Musculus/psychic?q=16:97207391-97207413) | **A**TC**A**T**T**GATGTTA**C**TACTGT | GGG | 4 | + | Intergenic |
| [18:19885222-19885244](http://www.ensembl.org/Mus_Musculus/psychic?q=18:19885222-19885244) | CT**AC**TAGAT**A**TTAGTACTG**G** | AGG | 4 | + | Intergenic |
| [18:22597253-22597275](http://www.ensembl.org/Mus_Musculus/psychic?q=18:22597253-22597275) | C**AA**GTAGAT**T**TTAGTA**T**TGT | TGG | 4 | - | Intergenic |
| [18:59920352-59920374](http://www.ensembl.org/Mus_Musculus/psychic?q=18:59920352-59920374) | C**A**CG**G**A**A**ATGT**A**AGTACTGT | TGG | 4 | + | Intergenic |
| [18:85276336-85276358](http://www.ensembl.org/Mus_Musculus/psychic?q=18:85276336-85276358) | CTC**T**TAGA**A**GTTAGTA**A**TG**G** | TGG | 4 | + | Intergenic |
| [19:5005558-5005580](http://www.ensembl.org/Mus_Musculus/psychic?q=19:5005558-5005580) | CT**G**G**A**AGA**G**GTTAGT**C**CTGT | GGG | 4 | - | Intergenic |
| [19:28766115-28766137](http://www.ensembl.org/Mus_Musculus/psychic?q=19:28766115-28766137) | CT**A**G**CC**GA**G**GTTAGTACTGT | GGG | 4 | + | Intergenic |
| [2:11212249-11212271](http://www.ensembl.org/Mus_Musculus/psychic?q=2:11212249-11212271) | C**G**C**T**TAGATGT**G**AG**A**ACTGT | AGG | 4 | - | Intronic |
| [2:74856534-74856556](http://www.ensembl.org/Mus_Musculus/psychic?q=2:74856534-74856556) | CTC**A**TA**T**ATGTTA**T**TACT**T**T | AGG | 4 | - | Intronic |
| [2:124839819-124839841](http://www.ensembl.org/Mus_Musculus/psychic?q=2:124839819-124839841) | **A**T**T**GTAGATG**CC**AGTACTGT | GGG | 4 | + | Intergenic |
| [3:137650587-137650609](http://www.ensembl.org/Mus_Musculus/psychic?q=3:137650587-137650609) | CTC**C**TAG**T**TG**C**TAG**A**ACTGT | GGG | 4 | - | Intergenic |
| [4:57634824-57634846](http://www.ensembl.org/Mus_Musculus/psychic?q=4:57634824-57634846) | C**AG**GTAGATGTTAGTA**T**TG**A** | TGG | 4 | + | Intronic |
| [5:104657776-104657798](http://www.ensembl.org/Mus_Musculus/psychic?q=5:104657776-104657798) | CTC**C**T**TT**ATGTTAGT**T**CTGT | GGG | 4 | + | Intergenic |
| [5:104841990-104842012](http://www.ensembl.org/Mus_Musculus/psychic?q=5:104841990-104842012) | CTC**C**T**TT**ATGTTAGT**T**CTGT | GGG | 4 | - | Intergenic |
| [5:151379787-151379809](http://www.ensembl.org/Mus_Musculus/psychic?q=5:151379787-151379809) | CT**G**G**AT**GATGTTAG**G**ACTGT | AGG | 4 | - | Intergenic |
| [6:35097568-35097590](http://www.ensembl.org/Mus_Musculus/psychic?q=6:35097568-35097590) | CT**AT**TA**T**ATGTTAGTACTG**A** | GGG | 4 | - | Intronic |
| [6:37334379-37334401](http://www.ensembl.org/Mus_Musculus/psychic?q=6:37334379-37334401) | CT**T**G**G**AGA**A**GT**C**AGTACTGT | TGG | 4 | - | Exonic |
| [6:96099236-96099258](http://www.ensembl.org/Mus_Musculus/psychic?q=6:96099236-96099258) | CTC**C**TAGATGTT**G**G**G**A**G**TGT | AGG | 4 | - | Intergenic |
| [6:129301521-129301543](http://www.ensembl.org/Mus_Musculus/psychic?q=6:129301521-129301543) | **A**T**T**G**G**AGATGT**C**AGTACTGT | GGG | 4 | - | Intergenic |
| [7:20272931-20272953](http://www.ensembl.org/Mus_Musculus/psychic?q=7:20272931-20272953) | **A**T**T**G**G**AGATGT**C**AGTACTGT | GGG | 4 | + | Intergenic |
| [7:20951583-20951605](http://www.ensembl.org/Mus_Musculus/psychic?q=7:20951583-20951605) | **A**T**T**G**G**AGATGT**C**AGTACTGT | GGG | 4 | + | Intergenic |
| [7:22268585-22268607](http://www.ensembl.org/Mus_Musculus/psychic?q=7:22268585-22268607) | **A**T**T**G**G**AGATGT**C**AGTACTGT | GGG | 4 | + | Intergenic |
| [7:77231127-77231149](http://www.ensembl.org/Mus_Musculus/psychic?q=7:77231127-77231149) | CT**AC**TAGATGTT**G**GTACT**A**T | AGG | 4 | - | Intergenic |
| [8:31082792-31082814](http://www.ensembl.org/Mus_Musculus/psychic?q=8:31082792-31082814) | C**CT**G**G**AGATGTTAGTA**T**TGT | TGG | 4 | + | Intergenic |
| [8:114336947-114336969](http://www.ensembl.org/Mus_Musculus/psychic?q=8:114336947-114336969) | CT**A**G**G**AGATG**GC**AGTACTGT | AGG | 4 | - | Intronic |
| [8:120268584-120268606](http://www.ensembl.org/Mus_Musculus/psychic?q=8:120268584-120268606) | CTCG**G**AGA**A**G**GA**AGTACTGT | GGG | 4 | + | Intronic |
| [9:117273999-117274021](http://www.ensembl.org/Mus_Musculus/psychic?q=9:117273999-117274021) | CT**A**GT**C**GATGTTAG**G**ACTG**G** | TGG | 4 | - | Intronic |
| [X:47194687-47194709](http://www.ensembl.org/Mus_Musculus/psychic?q=X:47194687-47194709) | CTCG**A**AGATG**A**TAG**G**ACT**A**T | GGG | 4 | - | Intergenic |
| [X:139114227-139114249](http://www.ensembl.org/Mus_Musculus/psychic?q=X:139114227-139114249) | CTCGT**G**GATG**A**TAGT**G**CTG**A** | TGG | 4 | - | Intergenic |

| **sgRNA D1** |  |  |  |  |  |
| --- | --- | --- | --- | --- | --- |
| **Location** | **Sequence** | **PAM** | **Mismatches** | **Strand** | **Type** |
| Original CRISPR | AGACAAATGCCTGTCCTGCG | TGG |  |  | Intronic |
| [10:60530894-60530916](http://www.ensembl.org/Mus_Musculus/psychic?q=10:60530894-60530916) | AGACAAATGCCTGTCCTGCG | TGG | 0 | - | Intronic |
| [4:9284865-9284887](http://www.ensembl.org/Mus_Musculus/psychic?q=4:9284865-9284887) | AGAC**C**AAT**T**CCTGTCCTGCG | AGG | 2 | + | Intronic |
| [9:97492519-97492541](http://www.ensembl.org/Mus_Musculus/psychic?q=9:97492519-97492541) | AGACAAATGCCTGTCCT**TG**G | TGG | 2 | + | Intronic |
| [10:13240189-13240211](http://www.ensembl.org/Mus_Musculus/psychic?q=10:13240189-13240211) | AG**T**CA**G**ATGCCTGTCCT**T**CG | AGG | 3 | - | Intronic |
| [17:7868298-7868320](http://www.ensembl.org/Mus_Musculus/psychic?q=17:7868298-7868320) | **G**GACAAATG**T**CTGTCCTGC**T** | TGG | 3 | - | Intergenic |
| [17:85193062-85193084](http://www.ensembl.org/Mus_Musculus/psychic?q=17:85193062-85193084) | AGACA**T**AT**TT**CTGTCCTGCG | TGG | 3 | + | Intronic |
| [2:60427462-60427484](http://www.ensembl.org/Mus_Musculus/psychic?q=2:60427462-60427484) | AGACAAAT**A**CCTGTC**A**TGC**C** | AGG | 3 | - | Intronic |
| [7:132592637-132592659](http://www.ensembl.org/Mus_Musculus/psychic?q=7:132592637-132592659) | AGAC**CC**ATGCCTGTCCTG**A**G | AGG | 3 | - | Intronic |
| [9:3538737-3538759](http://www.ensembl.org/Mus_Musculus/psychic?q=9:3538737-3538759) | AGACA**C**ATGCCT**C**TCCTG**T**G | AGG | 3 | - | Intronic |
| [9:88711581-88711603](http://www.ensembl.org/Mus_Musculus/psychic?q=9:88711581-88711603) | AGACAAAT**T**CCT**C**TCCTG**G**G | AGG | 3 | + | Intronic |
| [1:3916406-3916428](http://www.ensembl.org/Mus_Musculus/psychic?q=1:3916406-3916428) | AG**C**CA**C**ATGCCT**C**TCCTG**T**G | GGG | 4 | + | Intergenic |
| [1:10719581-10719603](http://www.ensembl.org/Mus_Musculus/psychic?q=1:10719581-10719603) | AG**C**CAA**C**T**T**C**T**TGTCCTGCG | AGG | 4 | - | Intronic |
| [1:13844461-13844483](http://www.ensembl.org/Mus_Musculus/psychic?q=1:13844461-13844483) | AGACAAAT**AA**CTGT**A**CTGC**C** | AGG | 4 | + | Intergenic |
| [1:16575797-16575819](http://www.ensembl.org/Mus_Musculus/psychic?q=1:16575797-16575819) | AGACAAATGCCTGT**GT**T**AA**G | TGG | 4 | + | Intronic |
| [1:16883254-16883276](http://www.ensembl.org/Mus_Musculus/psychic?q=1:16883254-16883276) | AGACA**C**ATGCCTG**G**CCT**T**C**A** | GGG | 4 | - | Intergenic |
| [1:21157455-21157477](http://www.ensembl.org/Mus_Musculus/psychic?q=1:21157455-21157477) | AGA**A**AAATGCCT**C**TC**A**TGC**C** | TGG | 4 | + | Intergenic |
| [1:40533214-40533236](http://www.ensembl.org/Mus_Musculus/psychic?q=1:40533214-40533236) | AG**C**CA**T**ATGCCT**C**TCCTG**T**G | GGG | 4 | + | Intronic |
| [1:59592682-59592704](http://www.ensembl.org/Mus_Musculus/psychic?q=1:59592682-59592704) | AGACAA**GGAT**CTGTCCTGCG | GGG | 4 | - | Intronic |
| [1:63521451-63521473](http://www.ensembl.org/Mus_Musculus/psychic?q=1:63521451-63521473) | AGACA**C**AT**C**CCTGTCCT**AT**G | GGG | 4 | + | Intronic |
| [1:70871261-70871283](http://www.ensembl.org/Mus_Musculus/psychic?q=1:70871261-70871283) | A**C**ACAA**C**T**T**CCTGTCCTGC**C** | AGG | 4 | - | Intronic |
| [1:97379329-97379351](http://www.ensembl.org/Mus_Musculus/psychic?q=1:97379329-97379351) | AG**C**CA**C**ATGCCT**C**TCCTG**T**G | GGG | 4 | + | Intergenic |
| [1:109761612-109761634](http://www.ensembl.org/Mus_Musculus/psychic?q=1:109761612-109761634) | AG**T**CA**C**ATGCCT**C**TCCTG**T**G | GGG | 4 | - | Intergenic |
| [1:131911344-131911366](http://www.ensembl.org/Mus_Musculus/psychic?q=1:131911344-131911366) | AGACAAATGCC**CT**TCCT**C**C**A** | GGG | 4 | + | Intronic |
| [1:134714277-134714299](http://www.ensembl.org/Mus_Musculus/psychic?q=1:134714277-134714299) | **CAG**CA**G**ATGCCTGTCCTGCG | TGG | 4 | + | Intronic |
| [1:141421701-141421723](http://www.ensembl.org/Mus_Musculus/psychic?q=1:141421701-141421723) | AG**C**CA**C**ATGCCT**C**TCCTG**T**G | GGG | 4 | + | Intergenic |
| [1:153428946-153428968](http://www.ensembl.org/Mus_Musculus/psychic?q=1:153428946-153428968) | A**C**A**A**AAATGCCT**T**TCCTGC**C** | AGG | 4 | + | Intronic |
| [1:158573808-158573830](http://www.ensembl.org/Mus_Musculus/psychic?q=1:158573808-158573830) | AGACAAA**C**G**T**CTGT**G**CTGC**T** | AGG | 4 | - | Intronic |
| [1:175021099-175021121](http://www.ensembl.org/Mus_Musculus/psychic?q=1:175021099-175021121) | A**AG**CAAATGCCTG**A**CCTGC**T** | GGG | 4 | - | Intergenic |
| [1:194555590-194555612](http://www.ensembl.org/Mus_Musculus/psychic?q=1:194555590-194555612) | AG**C**CA**T**A**A**GCCT**C**TCCTGCG | GGG | 4 | + | Intergenic |
| [10:42173923-42173945](http://www.ensembl.org/Mus_Musculus/psychic?q=10:42173923-42173945) | A**C**ACAAATGCCT**TC**CCTG**A**G | TGG | 4 | - | Intergenic |
| [10:42310190-42310212](http://www.ensembl.org/Mus_Musculus/psychic?q=10:42310190-42310212) | AGACA**G**ATGCCTG**G**C**A**TG**T**G | AGG | 4 | + | Intergenic |
| [10:75145033-75145055](http://www.ensembl.org/Mus_Musculus/psychic?q=10:75145033-75145055) | A**T**A**A**AAA**G**G**G**CTGTCCTGCG | TGG | 4 | - | Intronic |
| [10:77569738-77569760](http://www.ensembl.org/Mus_Musculus/psychic?q=10:77569738-77569760) | AG**C**CA**C**ATGCCT**C**TCCTG**T**G | GGG | 4 | + | Intergenic |
| [10:101937342-101937364](http://www.ensembl.org/Mus_Musculus/psychic?q=10:101937342-101937364) | AG**C**CA**C**ATGCCT**C**TCCTG**T**G | GGG | 4 | + | Intronic |
| [10:121207655-121207677](http://www.ensembl.org/Mus_Musculus/psychic?q=10:121207655-121207677) | AGACAA**C**TGCCTG**CT**CTGC**C** | TGG | 4 | + | Intergenic |
| [10:127567192-127567214](http://www.ensembl.org/Mus_Musculus/psychic?q=10:127567192-127567214) | A**C**AC**C**AATGCCTGTCCT**CA**G | AGG | 4 | + | Exonic |
| [10:127973713-127973735](http://www.ensembl.org/Mus_Musculus/psychic?q=10:127973713-127973735) | AG**GT**A**T**ATGCCTGTCCTGC**A** | TGG | 4 | - | Intergenic |
| [11:48926979-48927001](http://www.ensembl.org/Mus_Musculus/psychic?q=11:48926979-48927001) | AGACAAA**GA**CCT**T**TCCTG**A**G | AGG | 4 | - | Exonic |
| [11:52580125-52580147](http://www.ensembl.org/Mus_Musculus/psychic?q=11:52580125-52580147) | AG**C**CA**C**ATGCCT**C**TCCTG**T**G | GGG | 4 | - | Intergenic |
| [11:61754675-61754697](http://www.ensembl.org/Mus_Musculus/psychic?q=11:61754675-61754697) | AGACAAAT**T**CCTGT**AG**TG**A**G | AGG | 4 | + | Intronic |
| [11:83000465-83000487](http://www.ensembl.org/Mus_Musculus/psychic?q=11:83000465-83000487) | AG**C**CA**C**ATGCCT**C**TCCTG**T**G | GGG | 4 | - | Intergenic |
| [11:83025799-83025821](http://www.ensembl.org/Mus_Musculus/psychic?q=11:83025799-83025821) | AG**C**CA**C**ATGCCT**C**TCCTG**T**G | GGG | 4 | - | Intergenic |
| [11:95336365-95336387](http://www.ensembl.org/Mus_Musculus/psychic?q=11:95336365-95336387) | AGACAAATG**AG**TGTC**A**TG**T**G | AGG | 4 | + | Intergenic |
| [11:103632688-103632710](http://www.ensembl.org/Mus_Musculus/psychic?q=11:103632688-103632710) | A**T**ACAAATG**T**CT**C**TCCTGC**A** | AGG | 4 | - | Intronic |
| [11:120892619-120892641](http://www.ensembl.org/Mus_Musculus/psychic?q=11:120892619-120892641) | AG**T**CA**T**AT**CA**CTGTCCTGCG | AGG | 4 | - | Intronic |
| [12:14752762-14752784](http://www.ensembl.org/Mus_Musculus/psychic?q=12:14752762-14752784) | AG**C**CA**C**ATGC**T**TGTCCTG**T**G | GGG | 4 | - | Intergenic |
| [12:41352356-41352378](http://www.ensembl.org/Mus_Musculus/psychic?q=12:41352356-41352378) | AGAC**G**AAT**T**CCTG**C**CCTG**G**G | TGG | 4 | - | Intronic |
| [12:72668977-72668999](http://www.ensembl.org/Mus_Musculus/psychic?q=12:72668977-72668999) | AGA**G**AAAT**TG**CTGTCCTG**A**G | AGG | 4 | + | Intergenic |
| [12:81966589-81966611](http://www.ensembl.org/Mus_Musculus/psychic?q=12:81966589-81966611) | AGACAAA**A**GCCT**A**TC**T**T**C**CG | AGG | 4 | - | Intronic |
| [12:86503248-86503270](http://www.ensembl.org/Mus_Musculus/psychic?q=12:86503248-86503270) | AGACAAA**G**GCCTGT**G**CT**AG**G | AGG | 4 | + | Intronic |
| [12:116162138-116162160](http://www.ensembl.org/Mus_Musculus/psychic?q=12:116162138-116162160) | **T**GACAAA**G**GCCTGT**T**CTG**T**G | GGG | 4 | - | Intergenic |
| [13:7062148-7062170](http://www.ensembl.org/Mus_Musculus/psychic?q=13:7062148-7062170) | AG**TA**AAATGC**T**TGTCCTG**A**G | AGG | 4 | - | Intergenic |
| [13:14843369-14843391](http://www.ensembl.org/Mus_Musculus/psychic?q=13:14843369-14843391) | AG**C**CA**C**ATGCCT**C**TCCTG**T**G | GGG | 4 | + | Intergenic |
| [13:23695699-23695721](http://www.ensembl.org/Mus_Musculus/psychic?q=13:23695699-23695721) | A**C**ACAAATGCC**CC**TCCT**C**CG | GGG | 4 | + | Intergenic |
| [13:53832835-53832857](http://www.ensembl.org/Mus_Musculus/psychic?q=13:53832835-53832857) | AG**C**CA**C**ATGCCT**C**TCCTG**T**G | GGG | 4 | - | Intergenic |
| [13:65732962-65732984](http://www.ensembl.org/Mus_Musculus/psychic?q=13:65732962-65732984) | **G**GACAAATG**TG**TGTCCT**T**CG | TGG | 4 | + | Intergenic |
| [13:66193364-66193386](http://www.ensembl.org/Mus_Musculus/psychic?q=13:66193364-66193386) | **G**GACAAATG**TG**TGTCCT**T**CG | TGG | 4 | - | Intergenic |
| [13:69987896-69987918](http://www.ensembl.org/Mus_Musculus/psychic?q=13:69987896-69987918) | AGACA**G**ATGCCTG**A**C**A**T**C**CG | AGG | 4 | - | Intergenic |
| [13:83177464-83177486](http://www.ensembl.org/Mus_Musculus/psychic?q=13:83177464-83177486) | AGACAAATGCCTGT**AAA**GC**A** | TGG | 4 | + | Intergenic |
| [13:85772142-85772164](http://www.ensembl.org/Mus_Musculus/psychic?q=13:85772142-85772164) | AG**C**CA**C**ATGCCT**C**TCCTG**T**G | GGG | 4 | + | Intergenic |
| [14:10464664-10464686](http://www.ensembl.org/Mus_Musculus/psychic?q=14:10464664-10464686) | AGACA**C**A**C**GCCT**A**TCCTG**T**G | GGG | 4 | - | Intronic |
| [14:47435072-47435094](http://www.ensembl.org/Mus_Musculus/psychic?q=14:47435072-47435094) | A**A**ACAA**T**TGCCTG**G**CCTGC**T** | AGG | 4 | + | Intergenic |
| [14:70337956-70337978](http://www.ensembl.org/Mus_Musculus/psychic?q=14:70337956-70337978) | AG**C**CA**C**ATGCCTG**C**CCTGC**T** | AGG | 4 | - | Intronic |
| [14:72369730-72369752](http://www.ensembl.org/Mus_Musculus/psychic?q=14:72369730-72369752) | **C**GAC**C**A**G**T**T**CCTGTCCTGCG | AGG | 4 | - | Intergenic |
| [15:77914149-77914171](http://www.ensembl.org/Mus_Musculus/psychic?q=15:77914149-77914171) | AGACAAATGCCTG**G**CC**ACT**G | GGG | 4 | + | Intergenic |
| [15:95483239-95483261](http://www.ensembl.org/Mus_Musculus/psychic?q=15:95483239-95483261) | A**A**ACAA**C**TGCCTGTCC**A**GC**C** | TGG | 4 | + | Intronic |
| [16:7327569-7327591](http://www.ensembl.org/Mus_Musculus/psychic?q=16:7327569-7327591) | A**A**ACAA**C**TGCCTGTCCTG**GT** | GGG | 4 | + | Intronic |
| [16:8830554-8830576](http://www.ensembl.org/Mus_Musculus/psychic?q=16:8830554-8830576) | AGACAA**C**TGC**TC**GTCCTGC**T** | CGG | 4 | - | Exonic |
| [16:27207459-27207481](http://www.ensembl.org/Mus_Musculus/psychic?q=16:27207459-27207481) | AG**C**CA**C**ATGCCT**C**TCCTG**T**G | GGG | 4 | - | Intergenic |
| [16:32844766-32844788](http://www.ensembl.org/Mus_Musculus/psychic?q=16:32844766-32844788) | **T**GA**TG**AATGCCTGTCCT**C**CG | AGG | 4 | - | Intronic |
| [16:34064517-34064539](http://www.ensembl.org/Mus_Musculus/psychic?q=16:34064517-34064539) | AGA**A**A**G**AT**T**CCTGTCCTG**T**G | TGG | 4 | - | Intronic |
| [16:48616617-48616639](http://www.ensembl.org/Mus_Musculus/psychic?q=16:48616617-48616639) | AGACA**G**ATGCCTGTCC**ATA**G | TGG | 4 | + | Intronic |
| [17:7166761-7166783](http://www.ensembl.org/Mus_Musculus/psychic?q=17:7166761-7166783) | AGAC**C**AAT**C**C**A**TGTCCTGC**C** | TGG | 4 | + | Intergenic |
| [17:24244931-24244953](http://www.ensembl.org/Mus_Musculus/psychic?q=17:24244931-24244953) | AGACA**G**AT**TT**CTGTCCTGC**C** | TGG | 4 | - | Intronic |
| [17:25274200-25274222](http://www.ensembl.org/Mus_Musculus/psychic?q=17:25274200-25274222) | **T**GACAAA**A**GC**G**TGTCC**A**GCG | GGG | 4 | - | Intronic |
| [17:37239357-37239379](http://www.ensembl.org/Mus_Musculus/psychic?q=17:37239357-37239379) | AG**C**CA**C**ATGCCT**C**TCCTG**T**G | GGG | 4 | + | Intergenic |
| [17:42523770-42523792](http://www.ensembl.org/Mus_Musculus/psychic?q=17:42523770-42523792) | AGACA**G**A**A**GCCTGTCCT**CT**G | AGG | 4 | + | Intergenic |
| [17:44748195-44748217](http://www.ensembl.org/Mus_Musculus/psychic?q=17:44748195-44748217) | AG**C**CA**C**ATGCCT**C**TCCTG**T**G | GGG | 4 | - | Intronic |
| [17:47512691-47512713](http://www.ensembl.org/Mus_Musculus/psychic?q=17:47512691-47512713) | AGA**AT**AATGCCTGTC**T**TGC**C** | TGG | 4 | - | Intronic |
| [17:80255346-80255368](http://www.ensembl.org/Mus_Musculus/psychic?q=17:80255346-80255368) | AGACAAATG**T**CTGTCCT**TGA** | CGG | 4 | + | Intronic |
| [18:3514292-3514314](http://www.ensembl.org/Mus_Musculus/psychic?q=18:3514292-3514314) | AGAC**CC**A**A**GCCT**T**TCCTGCG | GGG | 4 | - | Exonic |
| [18:13445768-13445790](http://www.ensembl.org/Mus_Musculus/psychic?q=18:13445768-13445790) | AG**C**CAAATG**G**CTGTC**T**TGC**A** | AGG | 4 | + | Intergenic |
| [18:20665468-20665490](http://www.ensembl.org/Mus_Musculus/psychic?q=18:20665468-20665490) | A**C**ACAAAT**A**CC**A**GTCC**A**GCG | AGG | 4 | - | Exonic |
| [18:31659007-31659029](http://www.ensembl.org/Mus_Musculus/psychic?q=18:31659007-31659029) | AGACAA**G**TGCC**A**G**G**CCTG**T**G | TGG | 4 | + | Intronic |
| [18:62762336-62762358](http://www.ensembl.org/Mus_Musculus/psychic?q=18:62762336-62762358) | AGA**G**AA**G**TGCCTG**A**CCTG**A**G | AGG | 4 | - | Intergenic |
| [19:9640858-9640880](http://www.ensembl.org/Mus_Musculus/psychic?q=19:9640858-9640880) | AG**C**CA**C**ATGCCT**T**TCCTG**T**G | GGG | 4 | - | Intergenic |
| [19:22575279-22575301](http://www.ensembl.org/Mus_Musculus/psychic?q=19:22575279-22575301) | AGA**A**AAATGCCTGT**TA**TGC**T** | GGG | 4 | + | Intronic |
| [19:23615943-23615965](http://www.ensembl.org/Mus_Musculus/psychic?q=19:23615943-23615965) | AGA**TG**AATGCCTGTC**TC**GCG | GGG | 4 | + | Intronic |
| [19:37197006-37197028](http://www.ensembl.org/Mus_Musculus/psychic?q=19:37197006-37197028) | **G**GACA**C**ATGCCTGT**G**CTG**T**G | AGG | 4 | - | Intronic |
| [19:37197077-37197099](http://www.ensembl.org/Mus_Musculus/psychic?q=19:37197077-37197099) | **G**GACA**C**ATGCCTGT**G**CTG**T**G | AGG | 4 | - | Intronic |
| [19:42674691-42674713](http://www.ensembl.org/Mus_Musculus/psychic?q=19:42674691-42674713) | AG**C**CA**C**ATGCCT**C**TCCTG**T**G | GGG | 4 | - | Intergenic |
| [19:50289478-50289500](http://www.ensembl.org/Mus_Musculus/psychic?q=19:50289478-50289500) | **T**GACAAATGC**AG**GT**A**CTGCG | AGG | 4 | + | Intronic |
| [2:18390996-18391018](http://www.ensembl.org/Mus_Musculus/psychic?q=2:18390996-18391018) | AG**TA**AAATGCCT**C**TCCTG**A**G | TGG | 4 | + | Intronic |
| [2:23464339-23464361](http://www.ensembl.org/Mus_Musculus/psychic?q=2:23464339-23464361) | AG**C**CA**T**ATGCCT**C**TCCTG**T**G | GGG | 4 | + | Intergenic |
| [2:122151408-122151430](http://www.ensembl.org/Mus_Musculus/psychic?q=2:122151408-122151430) | AGAC**C**AA**G**GCCTGTCCT**A**C**C** | TGG | 4 | - | Intronic |
| [2:125001351-125001373](http://www.ensembl.org/Mus_Musculus/psychic?q=2:125001351-125001373) | **G**GACAAATG**G**CTGTCC**CT**CG | TGG | 4 | + | Intergenic |
| [2:125641543-125641565](http://www.ensembl.org/Mus_Musculus/psychic?q=2:125641543-125641565) | AG**C**CA**C**ATGCCT**C**TCCTG**T**G | GGG | 4 | + | Intronic |
| [2:152178984-152179006](http://www.ensembl.org/Mus_Musculus/psychic?q=2:152178984-152179006) | A**A**ACAA**G**T**T**CCTGTCCTGC**C** | AGG | 4 | - | Intergenic |
| [2:167020663-167020685](http://www.ensembl.org/Mus_Musculus/psychic?q=2:167020663-167020685) | **T**GACAAA**A**GCCTGTCCT**T**C**C** | AGG | 4 | + | Exonic |
| [2:168600223-168600245](http://www.ensembl.org/Mus_Musculus/psychic?q=2:168600223-168600245) | **T**GACA**T**ATGCCTG**G**CCTGC**C** | TGG | 4 | + | Intronic |
| [2:170130773-170130795](http://www.ensembl.org/Mus_Musculus/psychic?q=2:170130773-170130795) | AGACAA**TG**GCCTG**C**CC**G**GCG | CGG | 4 | - | Intronic |
| [2:173424928-173424950](http://www.ensembl.org/Mus_Musculus/psychic?q=2:173424928-173424950) | **G**GACAAATGCCT**TC**CCTGC**T** | CGG | 4 | - | Intergenic |
| [3:4223328-4223350](http://www.ensembl.org/Mus_Musculus/psychic?q=3:4223328-4223350) | AGA**A**AAAT**CT**CTGTCCTG**T**G | TGG | 4 | - | Intergenic |
| [3:31415248-31415270](http://www.ensembl.org/Mus_Musculus/psychic?q=3:31415248-31415270) | **T**G**C**CAAATGCCTGTCCT**TG**G | TGG | 4 | - | Intergenic |
| [3:68542851-68542873](http://www.ensembl.org/Mus_Musculus/psychic?q=3:68542851-68542873) | A**TG**CA**GG**TGCCTGTCCTGCG | GGG | 4 | + | Intronic |
| [3:70059418-70059440](http://www.ensembl.org/Mus_Musculus/psychic?q=3:70059418-70059440) | AG**C**CA**C**ATGCCT**C**TCCTG**T**G | GGG | 4 | - | Intergenic |
| [3:79772908-79772930](http://www.ensembl.org/Mus_Musculus/psychic?q=3:79772908-79772930) | **T**GACA**C**ATGCCTG**G**CCTG**A**G | TGG | 4 | + | Intergenic |
| [3:94656390-94656412](http://www.ensembl.org/Mus_Musculus/psychic?q=3:94656390-94656412) | AGACAAATGC**T**TGTC**TG**G**T**G | TGG | 4 | + | Intronic |
| [3:101787097-101787119](http://www.ensembl.org/Mus_Musculus/psychic?q=3:101787097-101787119) | AG**C**CA**C**A**A**GCCT**C**TCCTGCG | GGG | 4 | - | Intergenic |
| [3:114547373-114547395](http://www.ensembl.org/Mus_Musculus/psychic?q=3:114547373-114547395) | AG**C**CA**C**ATGCCT**C**TCCTG**T**G | GGG | 4 | - | Intergenic |
| [3:117656761-117656783](http://www.ensembl.org/Mus_Musculus/psychic?q=3:117656761-117656783) | AG**C**CA**C**ATGCCT**C**TCCTG**T**G | GGG | 4 | + | Intronic |
| [3:119165411-119165433](http://www.ensembl.org/Mus_Musculus/psychic?q=3:119165411-119165433) | AGA**A**AAATGC**AG**GTCCTG**G**G | AGG | 4 | - | Intronic |
| [3:148585617-148585639](http://www.ensembl.org/Mus_Musculus/psychic?q=3:148585617-148585639) | AGACA**C**ATG**AT**TGT**T**CTGCG | TGG | 4 | + | Intergenic |
| [4:3856987-3857009](http://www.ensembl.org/Mus_Musculus/psychic?q=4:3856987-3857009) | AGACA**TC**TGCCTG**G**CCT**T**CG | AGG | 4 | + | Intergenic |
| [4:7821643-7821665](http://www.ensembl.org/Mus_Musculus/psychic?q=4:7821643-7821665) | AGACA**TC**TGCCTGTCCT**TG**G | TGG | 4 | - | Intergenic |
| [4:57140405-57140427](http://www.ensembl.org/Mus_Musculus/psychic?q=4:57140405-57140427) | A**C**ACAA**G**TG**T**CTGTCCTG**A**G | AGG | 4 | + | Intronic |
| [4:74909623-74909645](http://www.ensembl.org/Mus_Musculus/psychic?q=4:74909623-74909645) | AG**C**CA**C**ATGCCT**C**TCCTG**T**G | GGG | 4 | + | Intergenic |
| [4:84112243-84112265](http://www.ensembl.org/Mus_Musculus/psychic?q=4:84112243-84112265) | AGACA**TT**TGC**T**TGTCCTGC**A** | AGG | 4 | - | Intergenic |
| [4:119639852-119639874](http://www.ensembl.org/Mus_Musculus/psychic?q=4:119639852-119639874) | AGACAAAT**T**CCTGTCCT**CGC** | TGG | 4 | - | Intergenic |
| [4:135891244-135891266](http://www.ensembl.org/Mus_Musculus/psychic?q=4:135891244-135891266) | AG**GT**AAATG**T**CTGTCCTG**A**G | AGG | 4 | - | Intergenic |
| [4:149208082-149208104](http://www.ensembl.org/Mus_Musculus/psychic?q=4:149208082-149208104) | AGA**GG**AA**A**GCCTGTCCTG**T**G | TGG | 4 | - | Intronic |
| [5:22448752-22448774](http://www.ensembl.org/Mus_Musculus/psychic?q=5:22448752-22448774) | AG**GG**AAATGCCTGT**G**CTGC**C** | TGG | 4 | + | Intergenic |
| [5:33459118-33459140](http://www.ensembl.org/Mus_Musculus/psychic?q=5:33459118-33459140) | AG**G**CAAATG**G**CTGTCCT**T**C**T** | AGG | 4 | + | Intergenic |
| [5:44652367-44652389](http://www.ensembl.org/Mus_Musculus/psychic?q=5:44652367-44652389) | AGACAAATGCCTGT**T**C**GA**C**A** | TGG | 4 | + | Intronic |
| [5:78700223-78700245](http://www.ensembl.org/Mus_Musculus/psychic?q=5:78700223-78700245) | AG**C**CA**C**ATGCCT**C**TCCTG**T**G | GGG | 4 | + | Intergenic |
| [5:83663391-83663413](http://www.ensembl.org/Mus_Musculus/psychic?q=5:83663391-83663413) | AGACAA**G**TGCCT**T**T**T**C**A**GCG | AGG | 4 | - | Intergenic |
| [5:86559754-86559776](http://www.ensembl.org/Mus_Musculus/psychic?q=5:86559754-86559776) | AGACAAAT**T**C**T**TGTCCT**T**C**T** | AGG | 4 | + | Intronic |
| [5:96333852-96333874](http://www.ensembl.org/Mus_Musculus/psychic?q=5:96333852-96333874) | AG**G**CAAAT**A**CCTGTCCTG**GA** | AGG | 4 | + | Intergenic |
| [5:104391990-104392012](http://www.ensembl.org/Mus_Musculus/psychic?q=5:104391990-104392012) | AG**C**CA**C**ATGCCT**C**TCCTG**T**G | GGG | 4 | + | Intergenic |
| [5:115704184-115704206](http://www.ensembl.org/Mus_Musculus/psychic?q=5:115704184-115704206) | AG**C**CA**C**ATGCCT**C**TCCTG**T**G | GGG | 4 | - | Intronic |
| [5:116478292-116478314](http://www.ensembl.org/Mus_Musculus/psychic?q=5:116478292-116478314) | AGA**T**AAATGCCTGTCCT**CAA** | CGG | 4 | - | Intronic |
| [5:142781563-142781585](http://www.ensembl.org/Mus_Musculus/psychic?q=5:142781563-142781585) | AGACA**G**ATG**T**CTGTCCT**T**C**C** | TGG | 4 | - | Intronic |
| [5:142792041-142792063](http://www.ensembl.org/Mus_Musculus/psychic?q=5:142792041-142792063) | A**TT**CA**C**ATGCCTGTCCTG**A**G | AGG | 4 | + | Intronic |
| [5:142792721-142792743](http://www.ensembl.org/Mus_Musculus/psychic?q=5:142792721-142792743) | AGACAAA**A**GCCT**TA**CCTG**T**G | TGG | 4 | - | Intronic |
| [5:143709800-143709822](http://www.ensembl.org/Mus_Musculus/psychic?q=5:143709800-143709822) | AGACA**GT**T**C**CCTGTCCTGC**T** | GGG | 4 | + | Exonic |
| [6:34439405-34439427](http://www.ensembl.org/Mus_Musculus/psychic?q=6:34439405-34439427) | **T**GACA**C**ATGCCTGT**T**CT**C**CG | TGG | 4 | + | Intergenic |
| [6:53335061-53335083](http://www.ensembl.org/Mus_Musculus/psychic?q=6:53335061-53335083) | A**A**ACAA**G**TGCCTGTC**T**TG**T**G | GGG | 4 | - | Intergenic |
| [6:53530871-53530893](http://www.ensembl.org/Mus_Musculus/psychic?q=6:53530871-53530893) | A**C**ACAAA**G**GCCT**T**T**T**CTGCG | TGG | 4 | + | Intergenic |
| [6:57399494-57399516](http://www.ensembl.org/Mus_Musculus/psychic?q=6:57399494-57399516) | AGACAAATGCCT**T**TC**T**T**CA**G | AGG | 4 | - | Intergenic |
| [6:60020019-60020041](http://www.ensembl.org/Mus_Musculus/psychic?q=6:60020019-60020041) | AG**C**CA**C**ATGCCT**C**TCCTG**T**G | GGG | 4 | - | Intergenic |
| [6:115931272-115931294](http://www.ensembl.org/Mus_Musculus/psychic?q=6:115931272-115931294) | AGACA**C**AT**T**CCTGTCC**A**G**G**G | AGG | 4 | - | Intergenic |
| [7:19072940-19072962](http://www.ensembl.org/Mus_Musculus/psychic?q=7:19072940-19072962) | AGA**AG**AATGCCTGT**T**CTGC**T** | GGG | 4 | - | Intronic |
| [7:19707677-19707699](http://www.ensembl.org/Mus_Musculus/psychic?q=7:19707677-19707699) | A**C**ACAA**C**TGCCTGTCCT**C**C**A** | AGG | 4 | - | Intronic |
| [7:67152656-67152678](http://www.ensembl.org/Mus_Musculus/psychic?q=7:67152656-67152678) | AGACAAA**A**GCC**A**GTCCT**CA**G | AGG | 4 | + | Intergenic |
| [7:81066489-81066511](http://www.ensembl.org/Mus_Musculus/psychic?q=7:81066489-81066511) | AGA**G**A**G**AT**C**CCTGTCCTG**G**G | TGG | 4 | + | Intronic |
| [7:133110426-133110448](http://www.ensembl.org/Mus_Musculus/psychic?q=7:133110426-133110448) | AGA**A**AA**C**TG**G**CTGTCCTGC**A** | GGG | 4 | - | Intronic |
| [7:142294741-142294763](http://www.ensembl.org/Mus_Musculus/psychic?q=7:142294741-142294763) | AGA**G**A**G**AT**C**CCTGTCCTGC**C** | TGG | 4 | + | Intergenic |
| [7:143122545-143122567](http://www.ensembl.org/Mus_Musculus/psychic?q=7:143122545-143122567) | **G**GACAAATG**G**CTGTC**A**TG**T**G | TGG | 4 | + | Intronic |
| [7:144971272-144971294](http://www.ensembl.org/Mus_Musculus/psychic?q=7:144971272-144971294) | AGACAA**C**TGCCTG**G**CCTG**GC** | AGG | 4 | - | Intergenic |
| [8:52775818-52775840](http://www.ensembl.org/Mus_Musculus/psychic?q=8:52775818-52775840) | AG**T**CA**C**ATGCCT**C**TCCTGC**A** | GGG | 4 | + | Intergenic |
| [8:72520600-72520622](http://www.ensembl.org/Mus_Musculus/psychic?q=8:72520600-72520622) | A**A**ACAAATGCCTGT**G**CTG**TA** | AGG | 4 | + | Intronic |
| [8:77729726-77729748](http://www.ensembl.org/Mus_Musculus/psychic?q=8:77729726-77729748) | AG**G**CAAAT**T**CCTGT**G**CTGC**T** | GGG | 4 | - | Intergenic |
| [8:79904182-79904204](http://www.ensembl.org/Mus_Musculus/psychic?q=8:79904182-79904204) | AG**C**CAA**C**TGCCT**T**TCCTGC**A** | GGG | 4 | - | Intergenic |
| [8:82493267-82493289](http://www.ensembl.org/Mus_Musculus/psychic?q=8:82493267-82493289) | AG**C**CA**C**ATGCCT**C**TCCTG**T**G | GGG | 4 | - | Intergenic |
| [8:94325295-94325317](http://www.ensembl.org/Mus_Musculus/psychic?q=8:94325295-94325317) | A**A**ACAAAT**C**CCTGTCCT**CT**G | GGG | 4 | - | Intergenic |
| [8:95227195-95227217](http://www.ensembl.org/Mus_Musculus/psychic?q=8:95227195-95227217) | AGACA**G**ATGCCT**C**T**T**CTG**T**G | TGG | 4 | - | Intergenic |
| [8:107682357-107682379](http://www.ensembl.org/Mus_Musculus/psychic?q=8:107682357-107682379) | AG**T**CAAATGCCTGTC**A**TG**GA** | TGG | 4 | - | Intergenic |
| [8:113433867-113433889](http://www.ensembl.org/Mus_Musculus/psychic?q=8:113433867-113433889) | A**TT**CAAATGCCT**C**TCCTG**A**G | AGG | 4 | + | Intergenic |
| [8:122573296-122573318](http://www.ensembl.org/Mus_Musculus/psychic?q=8:122573296-122573318) | AG**C**CA**C**ATGCCT**C**TCCTG**T**G | GGG | 4 | - | Intronic |
| [8:128421844-128421866](http://www.ensembl.org/Mus_Musculus/psychic?q=8:128421844-128421866) | AGAC**G**AA**G**G**T**CTGTCCTGC**C** | TGG | 4 | + | Intronic |
| [9:40820221-40820243](http://www.ensembl.org/Mus_Musculus/psychic?q=9:40820221-40820243) | **T**G**T**CAA**G**TGCCTGTCCT**T**CG | GGG | 4 | + | Intergenic |
| [9:43779483-43779505](http://www.ensembl.org/Mus_Musculus/psychic?q=9:43779483-43779505) | **G**GACAAATGCC**A**GTCCT**CG**G | TGG | 4 | + | Intronic |
| [9:56668732-56668754](http://www.ensembl.org/Mus_Musculus/psychic?q=9:56668732-56668754) | AG**C**C**C**AATGCCTG**A**CCTG**G**G | CGG | 4 | - | Intronic |
| [9:61382360-61382382](http://www.ensembl.org/Mus_Musculus/psychic?q=9:61382360-61382382) | AGA**A**AAATGCCT**CA**CCTGC**T** | TGG | 4 | - | Intronic |
| [9:65208406-65208428](http://www.ensembl.org/Mus_Musculus/psychic?q=9:65208406-65208428) | AG**T**CAAA**C**GCCT**C**TCCTG**T**G | GGG | 4 | - | Intergenic |
| [9:70196690-70196712](http://www.ensembl.org/Mus_Musculus/psychic?q=9:70196690-70196712) | AGACAA**G**TGCCTG**C**CCTG**AT** | GGG | 4 | - | Intergenic |
| [9:89219373-89219395](http://www.ensembl.org/Mus_Musculus/psychic?q=9:89219373-89219395) | AGACAAAT**T**CCT**CC**CCTG**G**G | TGG | 4 | - | Intronic |
| [9:109584740-109584762](http://www.ensembl.org/Mus_Musculus/psychic?q=9:109584740-109584762) | AG**C**CA**C**ATGCCT**C**TCCTG**T**G | GGG | 4 | + | Intergenic |
| [X:8073776-8073798](http://www.ensembl.org/Mus_Musculus/psychic?q=X:8073776-8073798) | AGAC**T**AAT**C**CCT**C**TCCT**C**CG | AGG | 4 | + | Intronic |
| [X:102628947-102628969](http://www.ensembl.org/Mus_Musculus/psychic?q=X:102628947-102628969) | AG**C**CA**C**ATGCCT**C**TCCTG**T**G | GGG | 4 | - | Intronic |
| [X:126368853-126368875](http://www.ensembl.org/Mus_Musculus/psychic?q=X:126368853-126368875) | AG**C**CA**C**ATGCCT**C**TCCTG**T**G | GGG | 4 | + | Intergenic |
| [X:139906956-139906978](http://www.ensembl.org/Mus_Musculus/psychic?q=X:139906956-139906978) | AGACAAAT**A**CC**A**GTCCT**C**C**C** | TGG | 4 | + | Intronic |
| [X:166896493-166896515](http://www.ensembl.org/Mus_Musculus/psychic?q=X:166896493-166896515) | AG**T**CA**C**ATGCCT**C**TCCTG**T**G | GGG | 4 | - | Intergenic |

Table S3. Oligonucleotide sequences for Sanger sequencing of sgRNA_U1 and sgRNA_D1 predicted off-target sites (≤3 mismatches)

| **sgRNA** | **Predicted off-target site*** | **Forward Oligo Sequence 5’-3’** | **Reverse Oligo Sequence 5’-3’** |
| --- | --- | --- | --- |
| U1 | 12:30168821-30168843 | CTCCCCGTGCTCTGTTCTTT | TGACAGGGAACTCTGGGGAA |
|  | 13:37743124-37743146 | GGAGCCCTGAAAACAATTCCG | CTTAGGTCATGGGAGACGGTG |
|  | 16:55439600-55439622 | AGGTGAGAAATTCTGGTGAGAAA | AGTGGGTTGCTTCCTGTCTT |
|  | 16:87306283-87306305 | GCTTCCTCGTCCAAGTGTGA | AAGGCATGACTGCTTTGCAC |
|  | 19:15387252-15387274 | AGCTCCTCTAATGACATACAAGCA | TCCGACCCTATAGCCCCATT |
| D1 | 4:9284865-9284887 | TCCAATGACTCATGAGCGAGA | TCAGGGCAAGATGGACGAAA |
|  | 9:97492519-97492541 | GTCCCAGGACGAGGAAGTGA | CGAGCTACAGCAGGCATACA |
|  | 10:13240189-13240211 | CTGTCTCTGGGGCAACTTGT | AACAGCCAGACTCCTTCTGC |
|  | 17:7868298-7868320 | TACTTTTGGCACCGTGTGGA | GTTGCAGTAGTAGCCGGTGT |
|  | 17:85193062-85193084 | ATCTGAATGGTCACGGGTTGT | GGAGTCTGAGCCTAGTGCAG |
|  | 2:60427462-60427484 | CTGGCAAACTCAACACCAGC | GAGTCGGCCTTTCTTGGTGA |
|  | 7:132592637-132592659 | CCTAAGTCTGACGGGTTAAG | GTAGAAATCACCAGCCTTCC |
|  | 9:3538737-3538759 | TCCTAGCTTTGGCTCATAAGTGT | ATTGTCCTATCAATGCATGACTCC |
|  | 9:88711581-88711603 | CACTGTGGCCATACTGGAAG | AAGCAGGAAGGAAGACAGGC |

*The location of the sgRNA predicted off-target sites relate to those detailed in Table S2.
